# Global landscape of protein-coding and long non-coding RNA alternative splicing and regulation in the human esophageal squamous cell carcinoma

**DOI:** 10.1186/s12935-025-03921-9

**Published:** 2026-06-22

**Authors:** Yunfei Wang, Jingkuan Yang, Shuling Li, Yuanfang Zhang, Jingwei Xue, Shengyu Su, Ziyu Liu, Zhiying Zhao, Zhikun Feng, Qiong Wu, Zigang Dong, Kangdong Liu, Yanan Jiang

**Affiliations:** 1 State Key Laboratory of Metabolic Dysregulation & Prevention and Treatment of Esophageal Cancer, School of Basic Medical Sciences, Zhengzhou University, Zhengzhou, 450000 Henan China; 2 The Pathophysiology Department, School of Basic Medical Sciences, Zhengzhou University, Zhengzhou, 450000 Henan China; 3 Tianjian Laboratory of Advanced Biomedical Sciences, Zhengzhou, 450000 Henan China; 4 China-US (Henan) Hormel Cancer Institute, No. 127, Dongming Road, Jinshui District, Zhengzhou, Henan China; 5 Provincial Cooperative Innovation Center for Cancer Chemoprevention, Zhengzhou University, Zhengzhou, 450000 Henan China; 6 Cancer Chemoprevention International Collaboration Laboratory, Zhengzhou, 450000 Henan China; 7 Department of Pathology, People’s Hospital of Zhengzhou, Zhengzhou, 450003 Henan China

**Keywords:** Alternative splicing, Splicing factor, Esophageal squamous cell carcinoma, Protein-coding RNA, LncRNA

## Abstract

**Background:**

Aberrant alternative splicing (AS) generates multiple isoforms from a single gene, leading to functional diversity and potentially contributing to tumorigenesis. The spliceosome pathway is significantly activated in esophageal squamous cell carcinoma (ESCC). However, the functional implications and drug-response relevance of protein-coding and long non-coding RNA (lncRNA) AS and splicing factors (SFs) have not been fully characterized in ESCC.

**Methods:**

The transcriptomic data (PRJNA533799, PRJNA435587, PRJNA704753, PRJNA489250, PRJNA298963) from Gene Expression Omnibus (GEO) database were used to summarize the AS landscape by rMATS analysis. Weighted correlation network analysis (WGCNA) and motif enrichment analysis were performed to investigate biological processes of AS events and SFs. The relationship between AS and drug sensitivity was explored by Spearman correlation analysis.

**Results:**

A total of 3699 differential alternative splicing events (DASEs) were identified, predominantly involving protein-coding RNA and lncRNA (96.65%). The DASEs and SFs were primarily involved in biological processes such as the cell cycle, immune response, metabolism, metastasis, and apoptosis. Moreover, 85 RNA binding proteins were identified as dysregulated and hub SFs to modulate AS in ESCC. Notably, Bortezomib and BI-2536 were sensitive in inhibiting ESCC cell proliferation, correlating strongly with the DASEs and SFs.

**Conclusion:**

Our research globally summarized the dysregulation of AS and SF, their associated biological effects, and recommended combined drug treatment strategies for ESCC. This study improves our knowledge of the molecular features of ESCC, supports the development of innovative therapies, and further advances fundamental research on precision medicine for ESCC.

**Supplementary Information:**

The online version contains supplementary material available at 10.1186/s12935-025-03921-9.

## Introduction

Esophageal cancer (ESCA), the sixth leading cause of cancer-related death, has two histological subtypes: esophageal adenocarcinoma (EAC) and esophageal squamous cell carcinoma (ESCC) [1]. Surgery, chemotherapy, and radiotherapy remain the primary treatment modalities for ESCC [2]. Furthermore, molecular features of omics-based analysis such as genomic, transcriptomic, proteomic, and epigenomic alterations have been studied to identify new treatment targets for ESCC [3–6]. However, the five-year survival rate still remains below 20% [7]. Therefore, it’s crucial to explore novel insights into the molecular characteristics of ESCC to discover therapeutic strategies.

Alternative splicing (AS), one of the posttranscriptional modifications in eukaryotes, plays a crucial role in increasing protein diversity and regulating physiological processes [8]. Aberrant AS is characterized as a hallmark of cancer as it can alter the function of translated proteins, potentially switching genes from tumor-suppressive to oncogenic isoforms and contributing to cancer development [9, 10]. The molecular features and functional consequences of AS have been characterized in multiple types of cancer, such as prostate cancer, gastric cancer and lung adenocarcinoma [11–13]. In ESCC, the spliceosome signaling pathway is significantly activated, and plenty of abnormal splicing events have been identified, indicating its imperative roles [5, 14–16]. However, the biological and functional implications of AS events in ESCC warrant further investigation.

Targeting AS represents a promising strategy for developing new cancer treatments [17, 18]. Splicing isoforms not only facilitate tumor advancement but also modulate the response to cancer therapies [19]. For example, aberrant splicing of folylpolyglutamate synthetase (FPGS) results in a loss of enzyme activity and subsequent resistance to methotrexate in leukemia [20]. Currently, splice-switching oligonucleotides (SSOs) are under investigation as potential therapeutic agents, with encouraging findings reported [21, 22]. Therefore, identifying key AS events or genes may present a diverse array of therapeutic targets. Despite this, the development of splice-related therapies for ESCC remains nascent.

Herein, we systematically summarize the AS landscape in ESCC using the transcriptomic data from Gene Expression Omnibus (GEO) database. Our analysis reveals extensive splicing disruptions in protein-coding RNA and lncRNA, as well as the identification of the dysregulated key splicing factors (SFs). Moreover, Bortezomib and BI-2536 are found to be sensitive to ESCC by potentially targeting AS or SFs. These findings shed new light on ESCC oncogenesis from the perspective of splicing and provide opportunities for innovative splicing-targeted strategies for ESCC.

## Methods and materials

### Data collection, processing, and differential expression analysis

We obtained the raw RNA-seq data for matched ESCC and adjacent tissue from GEO database (PRJNA533799, PRJNA435587, PRJNA704753, PRJNA489250, PRJNA298963). The raw sequence read archive (SRA) files were converted into FASTQ format using fastq-dump (v3.0.6) in SRA Toolkit (v3.0.6) and assessed for the quality with FastQC (v0.11.9), excluding any files that failed the ‘Per sequence quality scores’ or ‘Per base sequence quality’ from downstream analysis. Fastp (v0.23.2) with default settings was utilized to filter out low-quality reads. Then the clean reads were mapped to the human reference genome (GRCh38) using HISAT2 (v2.2.1). We quantified transcriptome abundance with featureCounts (v2.0.3) and applied variance stabilizing transformation (vst) in DESeq2 (v1.36.0) for normalization [23]. To confirm a robust result, we performed principal component analysis (PCA) clustering. After batch effect and outlier samples removal using removeBatchEffect function from the R package limma (v3.52.4), a 49 paired GEO ESCC meta cohort was obtained for downstream analysis. DESeq2 (v1.36.0) was employed to analyze the differential expression. Additionally, we gathered a microarray dataset of ESCC samples (GSE53625) to investigate the relationship between splicing factors and patient prognosis. We also obtained the raw proteomics data from two sources: the iProX database (IPX0002466000) [24], which includes 94 tumor tissues and 24 non-tumor esophageal tissues, and the PRIDE database (PXD021701) [5], which features paired tumor and adjacent non-tumor tissues from 124 EC patients.

### AS analysis

The rMATS tool (v4.0.2) [25] was used to identify splicing alterations for five main types of events: skipped exon (SE), alternative 5’ splice sites (A5SS), alternative 3’ splice sites (A3SS), mutually exclusive exons (MXE), and retained intron (RI). Significant AS events between tumor and normal tissue were defined by the following criteria: (i) false discovery rate (FDR) < 0.05; (ii) absolute inclusion level difference (|△PSI|) > 0.2; (iii) the sum of inclusion junction counts (IJC) and skipping junction counts (SJC) > 10; and (iv) occurrence in at least 8 pairs of samples. As a result, 3699 AS events were identified. AS events were visualized by the rmats2sashimiplot software (v2.0.4).

### Functional enrichment analysis

The R package WebGestaltR (v0.4.6) [26] was employed for enrichment analysis using over-representation analysis (ORA) and gene set enrichment analysis (GSEA) methods. Gene sets from Molecular Signatures Database (MSigDB) Hallmark (https://www.gsea-msigdb.org/gsea/msigdb/), Kyoto Encyclopedia of Genes and Genomes (KEGG), and Gene-ontology (GO) were utilized. *p* < 0.05 was considered significant for ORA method. FDR < 0.05 and|NES| >2.5 was considered significant for the GSEA method.

### Weighted Gene Co-Expression Network Analysis (WGCNA)

We adopted R package WGCNA (v1.72-1) [27] to identify significant modules correlated with ESCC. First, we computed the Pearson correlation coefficients for all gene across filtered samples based on the hierarchical clustering plot. These correlation values were then converted into an adjacency matrix using a soft thresholding of β = 8. The topological overlap matrix (TOM) was computed based on the adjacency matrix using the TOMsimilarity function. Then we applied hierarchical clustering to the TOM and got a hierarchical clustering tree of genes. Then, the clustering tree of genes was cut using a dynamic tree-cutting algorithm to make genes with similar expression patterns grouped into distinct modules, which were then assigned unique color labels for easy identification. Finally, a threshold of|r| >0.5 and *p* < 0.05 was set as the filter for module-trait correlation to obtain the valuable gene modules for subsequent analysis.

### Motif enrichment analysis

We employed the RNA Map Analysis and Plotting Server 2 (rMAPS2) [28] to characterize the RNA-binding proteins (RBPs). A control set of background exons was defined as those with FDR greater than 50%. The set of upregulated exons consisted of those with IncLevelDifference greater than 0, while the set of downregulated exons comprised exons with IncLevelDifference less than 0. A 250 bp intronic sequence in upstream and downstream of the exon-intron boundary and a 50 bp sequence at the 5’ and 3’ ends of the alternative exon were used to specify regions for motif enrichment analysis. By referring to the motif score calculated from rMAPS, we considered the position with the highest motif score as the most likely binding site for RBP. Motifs were considered significantly enriched if their p-value was less than 0.0005.

### Sensitivity estimation and discovery of splicing-associated drugs

Firstly, we employed the R package Oncopredict (v0.2) [29] to predict drug sensitivity. The training sets for drug prediction were obtained from three reputable resources: Cancer Therapeutics Response Portal (CTRP, http://portals.broadinstitute.org/ctrp.v2.1/), Genomics of Drug Sensitivity in Cancer (GDSC, https://www.cancerrxgene.org/), and the PRISM drug repurposing resource (https://depmap.org/repurposing/). Drug sensitivity was evaluated using the area under the dose-response curve (AUC), where lower values corresponded to greater sensitivity. Then, we constructed a ridge regression model to predict drug sensitivity. Following it, we compared the differences in sensitivity of these drugs between ESCC and adjacent samples and identified significantly sensitive drugs through strict criteria: FDR < 0.05 and log_2_(FoldChange)<−0.1. After that, Spearman correlation analysis was executed among drug sensitivity, AS events and SFs.

### Reagents and antibodies

The following antibodies and reagents are utilized in this study: SF3B2 (1:1000, Santa Cruz, sc-514930) and GAPDH (1:1000, Santa Cruz, sc-47724). JNK (1:500, SantaCruz, sc-7345), p-JNK (1:1000, Cell Signaling Technology, #4668). DMEM (VivaCell, C3110-0500), RPMI 1640 (VivaCell, C3010-0500), and fetal bovine serum (FBS) (VivaCell, C04001-0500), Trypsin-EDTA solution (Beyotime, C0201), penicillin-streptomycin solution (Sangon, E607011).

### Cell culture

SHEE cell line was generously gifted by Dr. Enmin Li of Shantou University. The KYSE30, KYSE70, KYSE140, KYSE150, KYSE410, KYSE450, and KYSE510 cell lines were purchased from the Academy of Science Cell Bank in Shanghai, China. The SHEE cell line was grown in DMEM supplemented with 10% FBS and 1% penicillin-streptomycin. The ESCC cell lines were maintained in RPMI-1640 medium supplemented with 10% FBS, 1% L-glutamine, and 1% penicillin-streptomycin.

### RT-PCR

Total RNA was isolated using TRIzol Reagent (Invitrogen,15596026). For cDNA synthesis, 1 µg of total RNA was reverse transcribed to cDNA with Takara PrimeScript RT reagent Kit with gDNA Eraser (Takara, RR047A). RT-PCR was conducted on 1µL of cDNA using 2x SanTaq PCR Master Mix (Sangon, B532061). PCR conditions were as described in the manufacturer’s protocol. PCR products were separated on 2% agarose gel. The list of primers used in this study is provided in Supplementary Table S1.

### Western blot

Protein lysates were subjected to sodium dodecyl sulfate (SDS)-polyacrylamide gel electrophoresis (PAGE), followed by transferring onto polyvinylidene fluoride (PVDF) membranes. The membranes were blocked with 5% skim milk for 1 h in TBS-Tween and incubated with primary antibodies at 4 °C overnight, and probed with antibodies against SF3B2 (1:1000, Santa Cruz, sc-514930), GAPDH (1:1000, Santa Cruz, sc-47724), JNK(1:500, SantaCruz, sc-7345), p-JNK(1:1000, Cell Signaling Technology, 4668), then incubated by a secondary goat anti-rabbit antibody (1:5000, ZSGB-BIO, ZB-2301) or a goat anti-mouse antibody (1:5000, ZSGB-BIO, ZB-2305) at room temperature for 1 h to 2 h. The targeted protein was visualized by electrochemiluminescence (ECL) luminescence reagent (Meilunbio, MA0186-1).

### Establishment of knockout SF3B2 ESCC cell lines

For CRISPR/Cas9-mediated knockout of human SF3B2, the sgRNA sequence was designed by the ChopChop website (https://chopchop.cbu.uib.no/). Then, DNA constructs and lentivirus packaging plasmids were transfected into HEK293T cells using jetPRIME® transfection reagent (Polyplus, 101000046/101000027). After 48 h and 72 h of transfection, virus-containing supernatants were collected and used to infect cells with 8 mg/mL polybrene. The transfected KYSE150 and KYSE450 cells were subjected to selection with 2 µg/mL puromycin for 3 days. The knockout efficiency of SF3B2 was detected by Western blot. The sgSF3B2 sequences used are in Supplementary Table S2.

### Rescue experiment

2 × 10^5^ KYSE150 cells were seeded and incubated for 16 h. When the cell density reaches 30%-40%, 10 µg of MAP2K7-S IRES-Puro-3×Flag plasmid was transfected. After transfection for 48 h, the transfection efficiency was validated by Western blot.

### Tissue microarray and immunohistochemical staining (IHC)

The ESCC tissue array containing 102 tumor tissues and 78 adjacent tissues, was purchased from Tufei Bio (Shanghai, China, TFeso-01), and was subjected to immunohistochemical staining. The sections were transferred to xylene solution and underwent dewaxing and hydration. Antigen retrieval was performed on the sections using high-temperature retrieval solution. 3% hydrogen peroxide (ORIGEN, PV-9000) was added and incubated for 20 min. Subsequently, the primary antibody (SF3B2, 1:50, Santa Cruz, sc-514930) was added and incubated overnight at 4 °C. Then, the appropriately diluted secondary antibody (Origene, ZF-0315, and ZT-0316) was added. The sections were processed with diaminobenzidine (DAB) solution (LABLEAD, D2004). A 0.1% hydrochloric acid alcohol differentiation solution was applied. Finally, the sections were imaged and quantitatively analyzed by Image-Pro Plus software. The integrated optical density (IOD) of all photos were calculated to evaluate the expression of SF3B2. According to the median expression of SF3B2, the patients were divided into the high SF3B2 and low SF3B2 expression groups.

### SiRNA transfection assay

SiRNA targeting SF3B2 (SF3B2#1, SF3B2#2) was synthesized by Sangon Biotech (Shanghai, China). The sequence is shown in Supplementary Table S2. 1 × 10^5^ cells were inoculated into a 6 cm dish treated with 20 µM of siRNA. After 48 h, Western blot was used to verify silencing efficiency.

### Cell proliferation assay

For the cell proliferation assay, 2 × 10^3^ KYSE150 were plated in 96 well plates and incubated for 0, 24, 48, 72, and 96 h, separately. The medium was supplemented with final concentration of 0.5 mg/mL methyl thiazolyl tetrazolium (MTT) at 37 °C for 2 h. Then, DMSO was added. OD value was performed at 490 nm with a microplate reader and analyzed with GraphPad software.

### Clone formation assay

KYSE150 cells were seeded at a density of 400 per well in 6 well plates. After 14 days, the clones were fixed by 4% polyoxymethylene (Beyotime, P0099). Visible colonies in each well were stained with 0.2% crystal violet at room temperature. Cell clones were counted and imaged using ImageJ and GraphPad software.

### Cytotoxicity assay

KYSE150 cells were seeded in 96-well plates at a density of 1 × 10^4^ cells per well and treated with a range of concentrations of BI-2536 (0,0.01,0.1,1,10µM), Bortezomib (0,0.01,0.1,1,10µM) for 24 and 48 h. The OD values of 490 nm were detected at 24 h and 48 h, respectively. IC50 values were calculated for both time points using GraphPad.

### Library preparation and RNA sequencing

The sgSF3B2 KYSE450 cells were collected, washed with phosphate-buffered saline (PBS), and then added 1mL Trizol. After being frozen in liquid nitrogen, it was sent for transcriptome sequencing (Novogene). In brief, RNA integrity was evaluated using the RNA Nano 6000 Assay Kit on the Bioanalyzer 2100 system (Agilent Technologies, CA, USA). Total RNA served as the input material for RNA sample preparation. mRNA was isolated from total RNA using poly-T oligo-attached magnetic beads. Fragmentation was performed with divalent cations in First Strand Synthesis Reaction Buffer (5X). First-strand cDNA synthesis was carried out using a random hexamer primer and M-MuLV Reverse Transcriptase (RNase H). Second-strand cDNA synthesis followed, using DNA Polymerase I and RNase H. Remaining overhangs were converted into blunt ends by exonuclease/polymerase activity. After adenylation of the 3’ ends of DNA fragments, hairpin loop adaptors were ligated to enable hybridization. To select cDNA fragments of about 370-420 bp, the library was purified using the AMPure XP system (Beckman Coulter, Beverly, USA). PCR amplification was done using Phusion High-Fidelity DNA polymerase, universal PCR primers, and an Index (X) Primer. Finally, PCR products were purified again (AMPure XP system), and library quality was assessed using the Agilent Bioanalyzer 2100 system. Indexed libraries were clustered using the cBot Cluster Generation System and the TruSeq PE Cluster Kit v3-cBot-HS (Illumina), following the manufacturer’s instructions. Sequencing was then conducted on the Illumina Novaseq platform, generating 150 bp paired-end reads.

The raw sequencing data (fastq format) were initially processed using in-house perl scripts. In this step, clean data (clean reads) were obtained by removing reads containing adapter, reads containing ploy-N and low quality reads from the raw data. At the same time, Q20, Q30 and GC content of the clean data were calculated. All the downstream analyses were based on the clean data with high quality. The subsequent steps including aligning the sequences to the reference genome, quantifying gene expression levels, differential expression analysis, and AS analysis were performed as previously described.

### Statistical analysis

The statistical analysis was performed using GraphPad Prism 8. All data were shown as the mean ± standard deviation (SD) and were compared using a Student’s t-test or one-way ANOVA. Survival analysis was performed using the Kaplan-Meier method with the log-rank test. Relationships between SFs and DASEs were analyzed Spearman correlation test. Bioinformatic statistical analyses were carried out using the default settings for each pipeline and portal. *p* < 0.05 was considered significant (*p < 0.05; **p < 0.01; ***p < 0.001).

## Results

### The landscape of differential alternative splicing events (DASEs) in ESCC

**Fig. 1:**
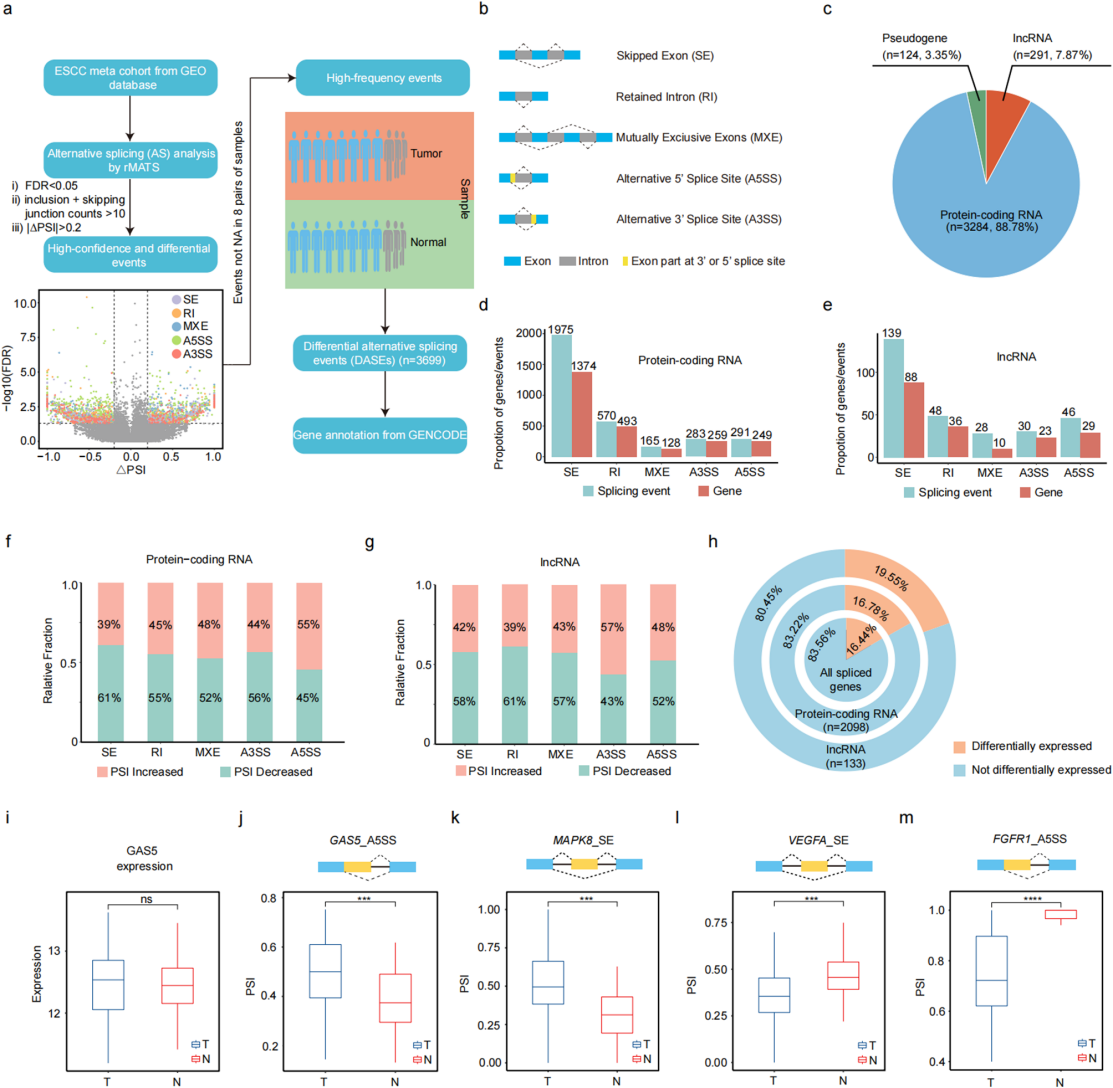
**The landscape of alternative splicing events and genes (DASEs) in ESCC.** **a** The workflow for identifying differentially alternative splicing events (DASEs) in ESCC. **b** Diagram of five different types of alternative splicing patterns. **c** Pie chart showing distribution of detected protein-coding, lncRNA and pseudogene splicing events. **d**,** e** Bar plot showing the number of DASEs and affected genes in protein-coding RNA (**d**) and lncRNA (**e**). **f**,** g** Bar plot showing the exonic inclusion level alterations of the five types of DASEs in protein-coding RNA (**f**) and lncRNA (**g**). **h** The proportion of differentially expressed (|Log_2_FC|>1, FDR < 0.05) and non-differentially expressed genes affected by DASEs. **i** Box plot showing the expression level of GAS5. **j** Box plot showing that PSI values for the GAS5 A5SS event. **k-m** Box plots showing the PSI values of *MAPK8* SE (**k**) *VEGFA* SE (**l**) *FGFR1* A5SS (**m**) events in ESCC samples compared to adjacent-normal samples, respectively.

To evaluate abnormal AS in ESCC, we followed the workflow diagram depicted in Fig. 1a. We collected RNA-seq data from GEO datasets and a 49 paired ESCC meta cohort was finally obtained. Principal components analysis (PCA) was conducted to assess the homogeneity and heterogeneity across all datasets (Supplementary Fig. 1a and b). Five splicing patterns were identified including SE, RI, A3SS, A5SS and MXE using rMATS analysis (Fig. 1b). Next, we filtered AS events in rMATS using strict criteria: FDR < 0.05, inclusion junction counts (IJC) + skipping junction counts (SJC) > 10,|△PSI|>0.2, and presence in at least 8 patients. Finally, we identified 3699 DASEs affecting 2305 genes (Supplementary Table S3). The proportion characteristics of 5 splicing types in ESCC patients was similar, with humble fluctuation of the number of DASEs (Supplementary Fig. 1c). 3284 (88.78%) were found in protein-coding RNAs, 291 (7.87%) in lncRNAs, and 124 (3.35%) in pseudogenes among DASEs (Fig. 1c). Thus, our primary focus was on splicing of protein-coding RNA and lncRNA. For protein-coding RNA, there were 1975 SE events in 1374 genes, 570 RI events in 493 genes, 283 A3SS events in 259 genes, 291 A5SS events in 249 genes and 165 MXE events in 128 genes (Fig. 1d). Similarly, for lncRNA, there were 139 SE events in 88 genes, 48 RI events in 36 genes, 30 A3SS events in 23 genes, 46 A5SS events in 29 genes and 28 MXE events in 10 genes (Fig. 1e). These results suggested that protein-coding RNA and lncRNA splicing mainly featured SE events and rarely MXE events.

Following this, we analyzed the exonic inclusion level alterations of the DASEs across 5 splicing types. For protein-coding RNA, downregulated PSI was observed in 61% of events in SE, 55% in RI, 52% in MXE, 56% in A3SS, and 45% in A5SS (Fig. 1f). For lncRNA, downregulated PSI was observed in 58% of events in SE, 61% in RI, 57% in MXE, 43% in A3SS, and 52% in A5SS (Fig. 1g). These results indicate that most splicing types of events show low exonic inclusion levels, except A5SS in protein-coding RNAs and A3SS in lncRNA in ESCC.

To evaluate the mRNA expression of spliced protein-coding RNA and lncRNA, we observed that only 352 spliced genes (16.78%) were differentially expressed in protein-coding RNA, while 26 spliced genes (19.55%) were differentially expressed in lncRNAs (Fig. 1h). These results indicated that assessing gene expression without considering splicing isoforms limits insights into the transcriptomic changes. For example, the long lncRNA GAS5 variant substantially promotes cell proliferation by regulating cell cycle arrest, while the short variant drives apoptosis in neuroblastoma [30]. The mRNA expression of the GAS5 gene showed no significant difference in ESCC compared to normal tissues (Fig. 1i). While the long GAS5 transcript exhibited a preferential expression in ESCC (Fig. 1j), indicating GAS5 may influence ESCC through AS.

It has been revealed that alterations of AS events are implicated in the effect of oncogenic, tumor-suppressive, and driver genes [31, 32]. By analyzing ESCC-related oncogenes, tumor suppressors, and driver genes using annotations derived from the CancerMine database [33], we found 9 driver genes (Supplementary Fig. 1d), 29 tumor suppressors (Supplementary Fig. 1e) and 45 oncogenes (Supplementary Fig. 1f) in DASEs-related genes (hypergeometric test, *p* = 5.18e-05, 3.94e-08 and 1.02e-13, respectively). Remarkably, these oncogenes were significantly enriched in oncogenic signaling pathways such as the MAPK, Ras, and Rap1 signaling pathways, as well as prostate cancer and renal cell carcinoma (Supplementary Fig. 1g). MAPK8, VEGFA, and FGFR1 are well-known factors functionally sustaining the MAPK signaling pathway, a critical pathway in the metastasis, differentiation, and survival of tumor [34]. ESCC exhibited a preferential expression of a long MAPK8 transcript (Fig. 1k), a short VEGFA transcript (Fig. 1l), and a short FGFR1 transcript (Fig. 1m) through AS. These results suggest that AS plays a role in activating oncogenic signaling pathways and promoting tumorigenesis in ESCC. Our findings reveal that AS events are dysregulated and may exert oncogenic effects on ESCC.

### Spliced genes and DASEs exhibit diverse splicing patterns and features

**Fig. 2:**
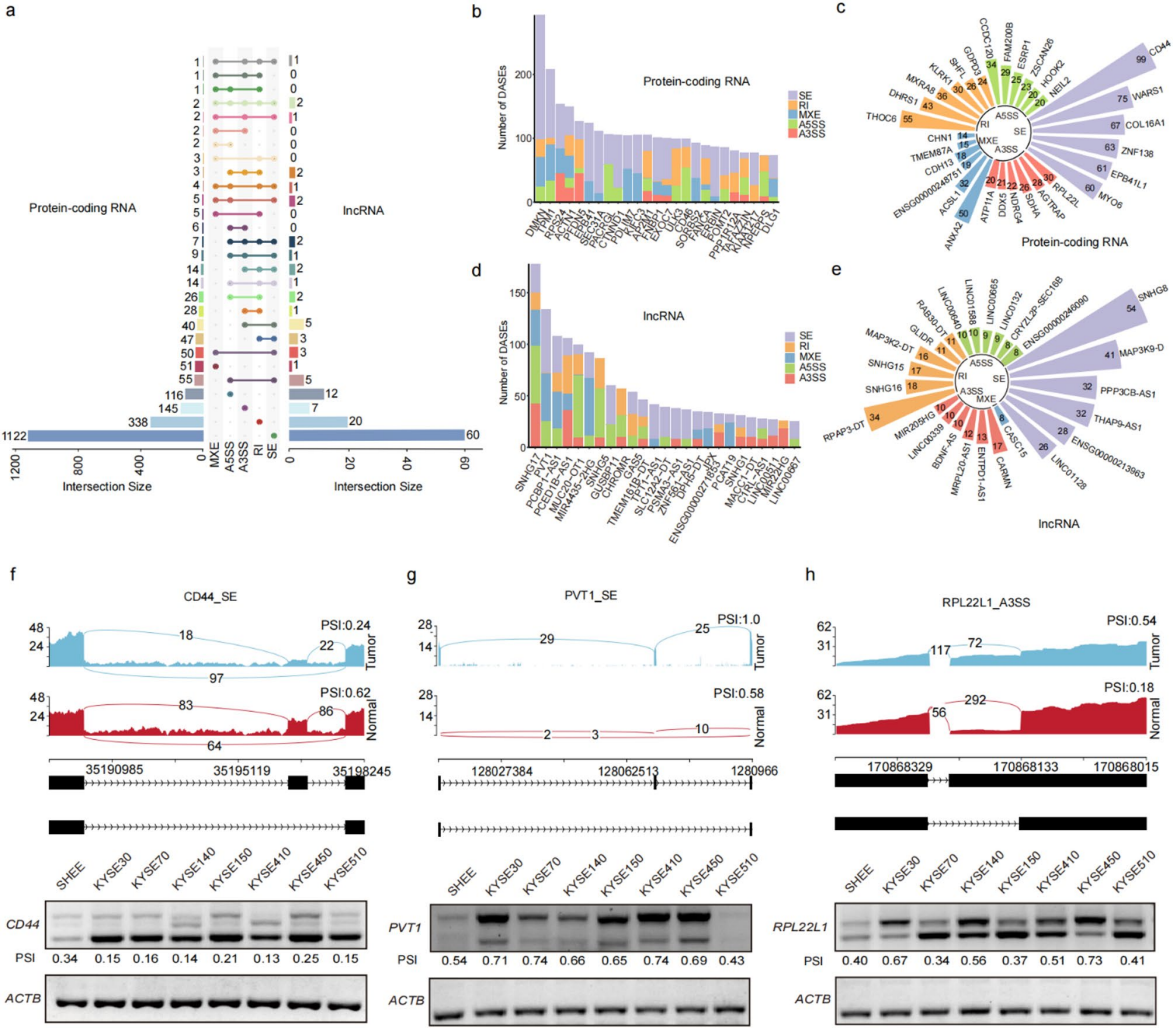
Spliced genes and DASEs exhibit diverse characteristics. **a** Upset plot illustrating the overlap among five types of DASEs in ESCC. **b-e** Bar plots depicting the ranking of protein-coding RNAs most affected by DASEs (**b, c**). Bar plots depicting the ranking of lncRNAs most affected by DASEs (**d, e**). **f-h** Sashimi plots and RT-PCR analyses of splicing events in CD44 (**f**), PVT1 (**g**) and RPL22L1 (**h**).

Next, we examined the splicing patterns of spliced genes and DASEs. Our analysis revealed that the majority of genes were disturbed by just one splicing type, with SE being the most prevalent (Fig. 2a). Notably, Dermokine (*DMKN*) and Tropomyosin 1 (*TPM1*) were the most frequently affected protein-coding genes (Fig. 2b). Moreover, the genes Cluster of Differentiation 44 (*CD44*) and Tryptophanyl-tRNA Synthetase 1 (*WARS1*), THO Complex Subunit 6 (*THOC6*) and Dehydrogenase/reductase 1 (*DHRS1*), Annexin A2 (*ANXA2*) and Acyl-CoA Aynthetase Long Chain family member 1 (*ACSL1*), coiled-coil domain containing 120 (*CCDC120*) and family with Sequence Similarity 200 Member B (*FAM200B*), Ribosomal Protein L22 Like 1 (*RPL22L1*), and Angiotensin II Receptor Associated Protein (*AGTRAP*) were the top-ranked genes in SE, RI, MXE, A5SS, and A3SS, respectively (Fig. 2c). For lncRNAs, Small Nucleolar RNA Host Gene 17 (*SNHG17*) and Plasmacytoma Variant Translocation 1 (*PVT1*) were the most frequently affected (Fig. 2d). Small Nucleolar RNA Host Gene 8 (*SNHG8*), RNA Polymerase II Associated Protein 3-Divergent transcript (*RPAP3-DT*), Cancer Susceptibility 15 (*CASC15*), Long Intergenic Non-Protein Coding RNA 640 (*LINC00640*) and Long Intergenic Non-Protein Coding RNA 1588 (*LINC01588*), Cardiac Mesoderm Enhancer-Associated Non-Coding RNA (*CARMN*) were the top-ranked genes in SE, RI, MXE, A5SS, and A3SS, respectively (Fig. 2e). Followingly, exon 10 skipping of *CD44*, exon 7 inclusion of *PVT1*, and A3SS in exon 3 of *RPL22L1* were confirmed by RT-PCR (Fig. 2f-h). These findings demonstrate spliced genes and DASEs display diverse splicing patterns and features in ESCC.

### The biological roles of DASEs in ESCC

**Fig. 3:**
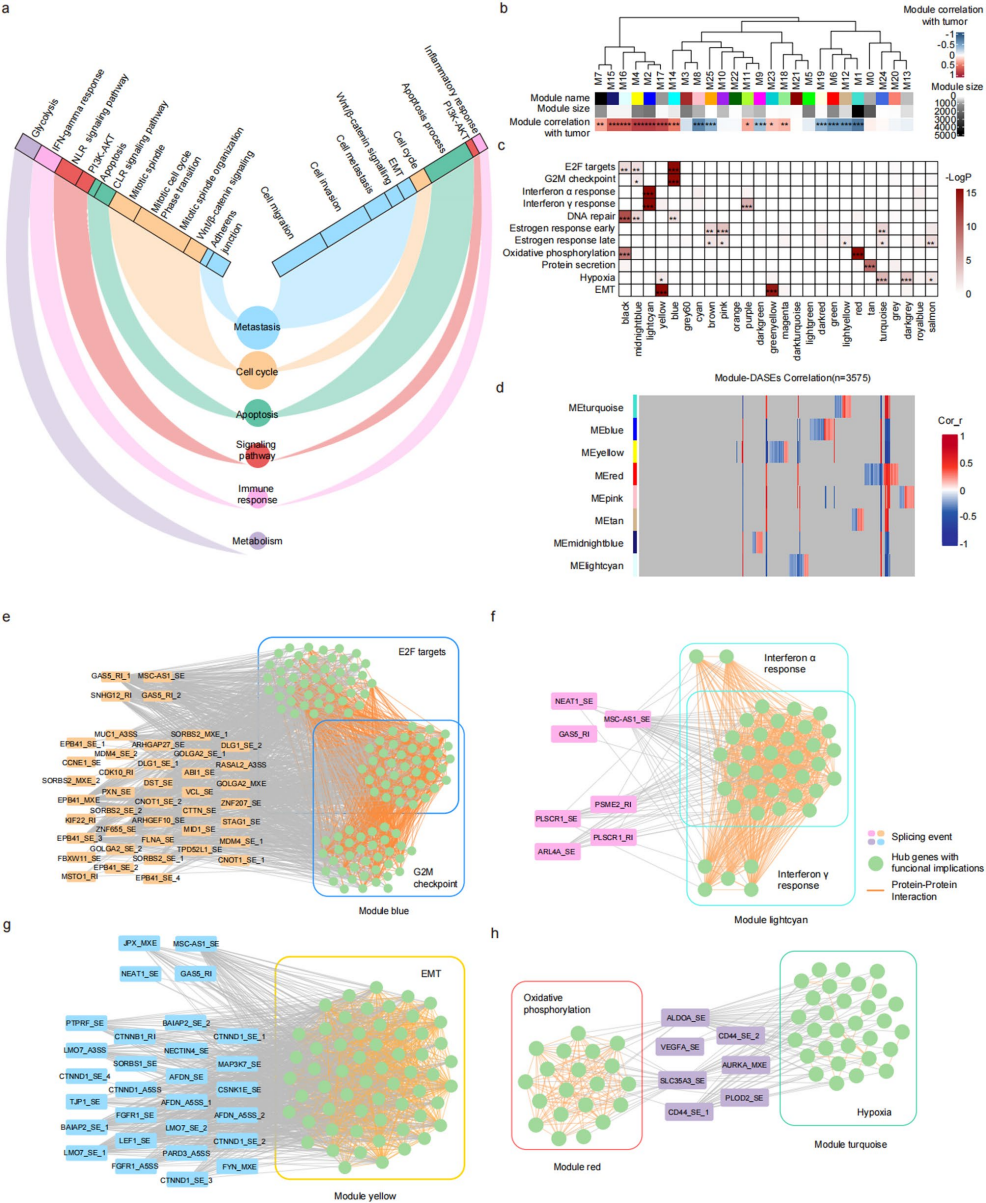
The biological process of DASEs in ESCC. **a** Hive plot illustrating pathways and corresponding biological processes (middle) associated with spliced protein-coding (left) and lncRNA (right) genes. **b** WGCNA describing the basic information of each module and characterizing its relationship with ESCC. **c** Heatmap of correlation between cancer-related hallmarks and all modules. **d** Heatmap illustrating the correlation between ME and DASEs. **e-h** Network diagram illustrating splicing events and hallmark-related hub genes interactions (FDR < 0.05,|r|>0.3). We first considered hallmark-related hub genes applying strict criteria: *(i)*|GS|>0.5,|MM|>0.5 and *(ii)* genes contained in hallmark signature

Uncovering the functional consequences of DASEs can discover clinically relevant AS alterations for therapy target [35]. We performed GO, KEGG, and Hallmark enrichment analyses on DASEs-related protein-coding genes. Our results indicated that most splicing types were associated with metastasis-associated processes such as the adherens junction. Specifically, genes with SE splicing exhibited significant enrichment in mitotic spindle and glycolysis, while DNA repair and immune response in RI, UV response and complement in MXE, PI3K/AKT/mTOR signaling pathway, DNA repair in A5SS, and heme metabolism in A3SS (Supplementary Fig. 2a). They were clustered into 6 critical biological processes: cell cycle, signaling pathway, apoptosis, metabolism, metastasis and immune response (Supplementary Fig. 2b). Of note, all spliced lncRNA genes were similarly enriched in these dimensions from LncSEA database (http://bio.liclab.net/LncSEA/index.php) [36] (Supplementary Fig. 2c). These results globally depicted functional consequences of spliced protein-coding and lncRNA genes in ESCC, categorized into 6 critical biological processes: cell cycle, signaling pathway, apoptosis, metabolism, metastasis and immune response (Fig. 3a and Supplementary Table S4). Overall, DASEs appear to significantly influence ESCC through critical biological processes of cancer hallmarks.

Numerous studies have proved that constructing expression networks to build relationships among protein-coding RNA, lncRNAs and isoform contributes to interrogating their functional implications [37–39]. Here, we carried out WGCNA using the transcriptome of protein-coding genes (Supplementary Fig. 3a-c). 26 modules were finally identified and labeled in ascending order of size from M0 to M25 with a different color (Fig. 3b and Supplementary Table S5). Module midnightblue, lightcyan, yellow, blue, grey60 were positively correlated with tumor (*r* > 0.5, *p* < 0.05), while red, tan, turquoise, and pink exhibited a strongly negative correlation (*r*<−0.5, *p* < 0.05). Therefore, we focused on the 9 cancer-associated modules. 8 of the 9 cancer-associated modules had at least one enriched hallmark process. The most significantly enriched hallmark processes were designated as the functional signatures of each module: blue represent E2F targets and G2/M checkpoint; lightcyan indicated the interferon response; red corresponded to oxidative phosphorylation; yellow indicated epithelial-mesenchymal transition (EMT); midnightblue corresponded to DNA repair; tan represent protein secretion; pink indicated the estrogen response and turquoise corresponded to hypoxia (Fig. 3c and Supplementary Table S6). Subsequently, we developed a module eigengene (ME) for each identified module and evaluated the relationship between these modules and DASEs. After filtering correlation pairs based on correlation coefficient (|r| >0.3) and FDR < 0.05, each ME was significantly associated with 545–958 DASEs (Fig. 3d and Supplementary Table S7). These findings highlight the correlation among DASEs, ME, and hallmark signature, providing novel insight into grasping the functional impact of DASEs on ESCC.

Notably, there was an evident overlap of functional consequences between gene- and splicing-level, except for apoptosis and signaling pathways that were not identified as a signature in WGCNA. To further consolidate the relationship between DASEs and hallmarks, we concentrated on DASEs associated with potential functions both in gene- and splicing-level. As a result, 42 DASEs were significantly correlated with cell cycle (Fig. 3e), 7 DASEs with immune response (Fig. 3f), 29 DASEs with metastasis (Fig. 3g), and 7 DASEs with metabolism (Fig. 3h). For DASEs affecting apoptosis and signaling pathways, consistent with expectations, there was a strong correlation between them (Supplementary Fig. 2d and e). These findings suggest that AS is involved in the cell cycle, signaling pathway, apoptosis, metabolism, metastasis, and immune response in ESCC.

### The biologically regulatory characteristics of splicing factors in ESCC

**Fig. 4:**
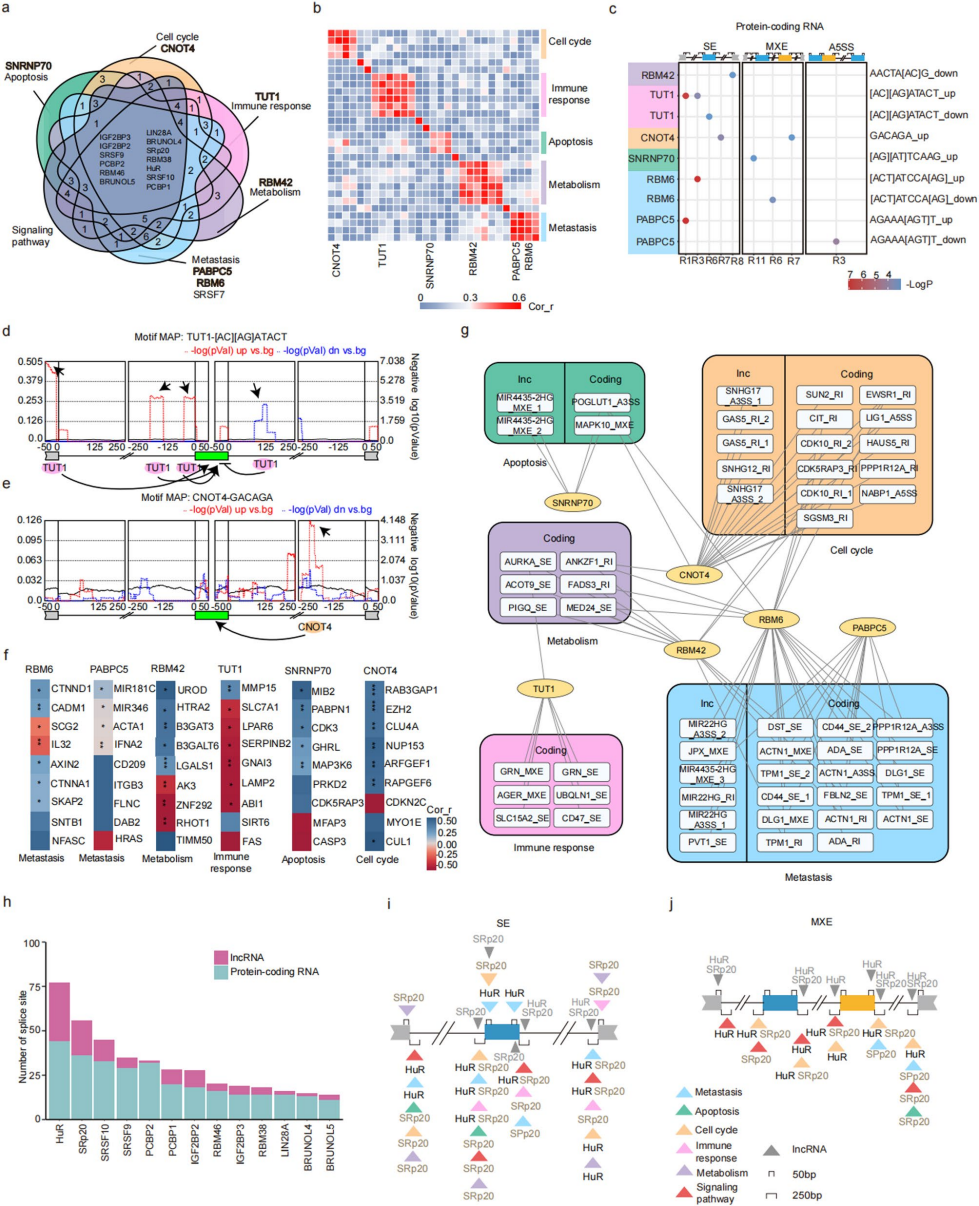
The biologically regulatory characteristics of splicing factors. **a** Venn diagram showing 7 exclusive SFs and 13 common SFs in 6 groups of SFs, along with their impacted biological functions. **b** Heatmap showed specific correlations between certain SFs and genes involved in particular biological processes. **c** Dot plot illustrating the distribution of cis-regulatory motifs across protein-coding splicing events. **d-e** Motif enrichment analysis of binding sites for TUT1 (**d**) and CNOT4 (**e**) surrounding alternatively spliced exons. Arrows mark peaks of significant motif over-enrichment. **f** Heatmap showing correlation between RBPs expression and genes associated with various biological processes. **g** Network of function-specific SFs and DASEs. **h** Bar plot displaying the number of splice sites for 13 shared SFs. **i-j** A comprehensive visualization of the distribution and regulatory impact of HuR and SRp20 in SE (**i**) and MEX (**j**)

Splicing factors drive the pathological AS on different hallmarks of cancer [12, 40]. Following that, we identified the splicing regulators responsible for the hallmark-related DASEs. RBPs regulate AS by binding specific sequences on the pre-mRNA (Supplementary Fig. 4a). To investigate the biological implications of splicing factors regulating DASEs, we performed RBP-binding motif enrichment analysis for the sequences flanking the differential exons, comparing against the background exons, in the regions around splicing sites (*p* < 0.0005). As a result, 59 RBPs were enriched in signaling pathway, 60 RBPs in cell cycle, 60 RBPs in immune response, 64 RBPs in metastasis, 42 RBPs in metabolism and 46 RBPs in apoptosis. To further discover function-specific and shared splicing factors, we first overlapped 6 groups of RBPs and found 7 exclusive RBPs (PABPC5, RBM6, SRSF7, RBM42, TUT1, CNOT4 and SNRNP70) and 13 shared RBPs (IGF2BP3, IGF2BP2, SRSF9, PCBP2, RBM46, HuR, PCBP1, LIN28A, BRUNOL4, SRp20, RBM38, BRUNOL5, SRSF10) (Fig. 4a). Furthermore, SRSF7 was significantly enriched in both blue (metastasis) and purple (metabolism) motifs (Supplementary Fig. 4b), whereas PABPC5 was significantly enriched only in blue (metastasis) (Supplementary Fig. 4c). RBM6, RBM42, SNRNP70, TUT1, and CNOT4 were significantly enriched in only one process motif (Supplementary Fig. 4d-h). Ultimately, we found motifs for PABPC5, RBM6, RBM42, CNOT4, TUT1, and SNRNP70 specifically enriched in exonic or intronic regions near the spliced exons with functional implications. Strikingly, dissection based on the expression relationship between RBPs and genes featured with functional impact showed significant specificity. In concrete terms, PABPC5 and RBM6 were implicated in metastasis, RBM42 in metabolism, CNOT4 in the cell cycle, TUT1 in immune response, and SNRNP70 in apoptosis (Fig. 4b). These data identify that RBPs specially regulate hallmark-related biological processes.

Next, we further explored the regulatory characterization of these SFs. Most of the cis-regulatory sequences regulated by motifs were included in SE type for protein-coding RNA (Fig. 4c). In contrast, they were evenly distributed in SE, MXE, A5SS, and A3SS for lncRNA (Supplementary Fig. 4i). To illustrate concretely, positional distribution of motifs showed that TUT1 recognition sequences ([AC][AG]ATACT) was the RBP-binding region for up- and down-regulated DASEs impacting immune response (Fig. 4d). CNOT4 recognition sequences (GACAGA) was the RBP-binding region for up-regulated DASEs impacting cell cycle, while the downregulated DASEs were not, suggesting a specific requirement for CNOT4 on the inclusion of its target DASEs (Fig. 4e). We also examined the data for any evidence of a regulatory relationship between RBP level and biological functions. These function-related RBPs was significantly correlated with the expression level of phenotypes-related genes (Fig. 4f). Similarly, RBPs were significantly correlated with the level of exon inclusion for DASEs with functional consequences. A correlation network was built using significantly correlated RBP-DASE pairs (|r|>0.3, FDR < 0.05) (Fig. 4g and Supplementary Table S8). These findings reveal the regulatory characterization of PABPC5, RMB6, RBM42, TUT1, CNOT4, and SNRNP70.

Following this, we focused on 13 shared RBPs to dissect their regulatory characterization. Of note, there were distinctive differences in the number of splice sites for 13 shared RBPs, and HuR and SRp20 were the RBPs harboring the most significant number of splice sites (Fig. 4h). So, we next investigated the regulatory characteristics of HuR and SRp20. Intriguingly, MXE and SE contained the most of splice site. For SE, motifs at intronic positions were evenly distributed across the 6 biological processes, while skipped exon positions were predominantly associated with metastasis and cell cycle, upstream and downstream exon positions mainly included metabolism and immune response, whereas lncRNAs were mainly around skipped exons (Fig. 4i). For MXE, motifs in intronic positions showed a marked enrichment for signaling pathway and cell cycle, whereas exon positions exhibited little to no enrichment for biological processes (Fig. 4j). Overall, we identify several SFs with notable biological implications and explore their regulatory characteristics.

### Dysregulated and hub splicing factors regulate DASEs and predict poor prognosis

**Fig. 5:**
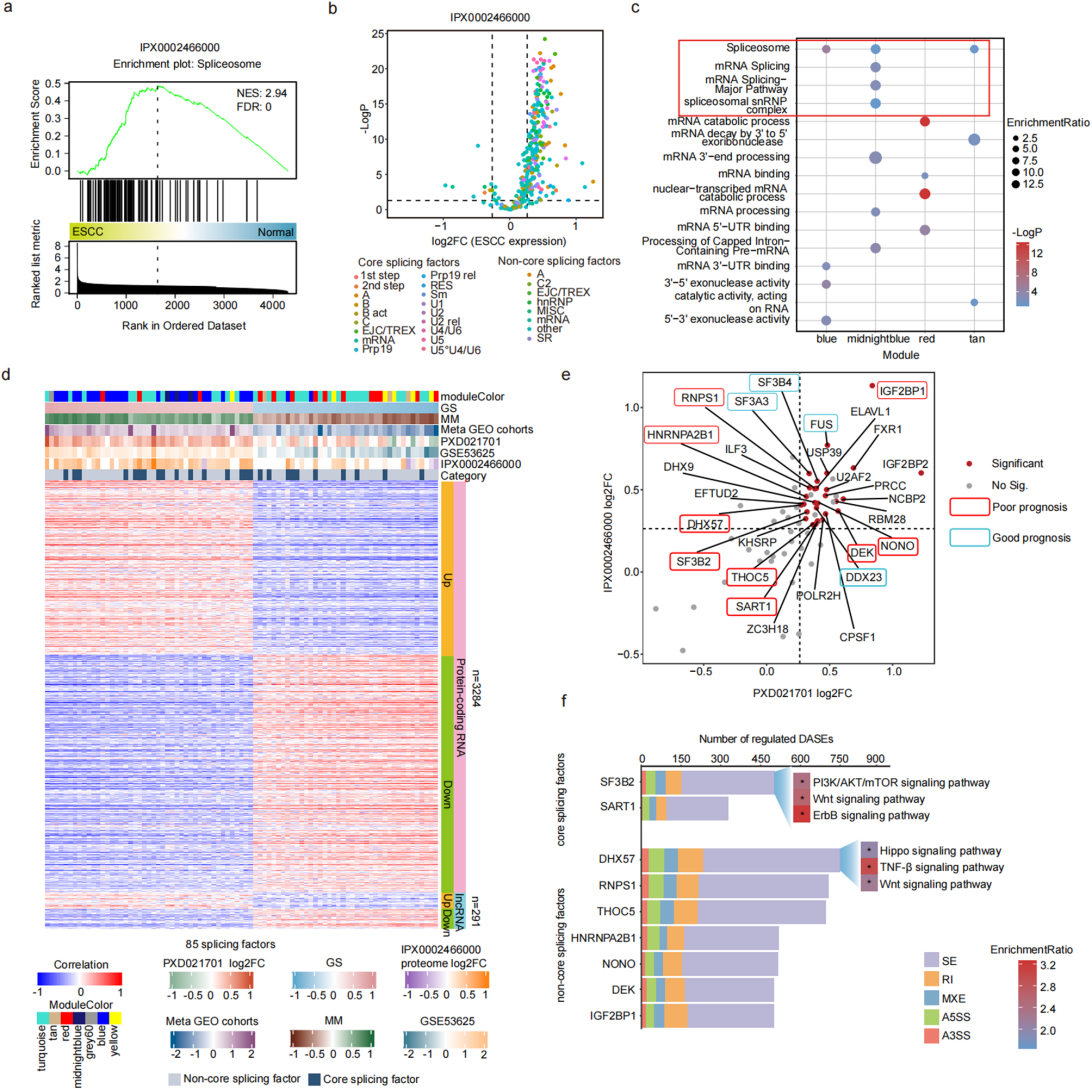
Dysregulated and hub splicing factors regulate DASEs and predict poor prognosis. **a** GSEA revealing splicesome pathway were significantly upregulated in ESCC. **b** Volcano plot showing SF expression levels in ESCC (FC > 1.2 or < 0.83, *p* < 0.05). **c** Functional enrichment analysis of key WGCNA modules showed pathways associated with alternative splicing. **d** Heatmap showing correlations between 85 SFs and DASEs. Columns represent SFs, and rows represent DASEs. **e** Scatter plot comparing SF expression in 2 proteomic cohorts. **f** Bar plot showing the number of different types of DASEs regulated by 9 SFs associated with poor prognosis. Functional enrichment results for SF3B2 and DHX57 are displayed on the right.

SFs directly bind pre-mRNA and regulate AS in a concentration-dependent manner [9]. Thus, defining the regulation landscape at SF levels is essential to comprehending SF-mediated transformation and advanced splicing-targeted therapies [41]. Using a list of 518 RBPs with an annotated role in AS [42, 43] (Supplementary Fig. 5a), we explored the protein expression of SFs. The spliceosome pathway was positively and significantly activated in ESCC (Fig. 5a) and ESCA (Supplementary Fig. 5b) by proteomic datasets (IPX0002466000 and PXD021701). Among the SFs that were quantifiable, 232 proteins of SFs exhibited significant expression differences, with 224 upregulated and 8 downregulated in ESCC, respectively (Fig. 5b). Similarly, 231 proteins of SFs significantly exhibited expression differences, with 218 upregulated and 13 downregulated in ESCA, respectively (Supplementary Fig. 5c). The data show that the expression of SFs is generally elevated in ESCC.

A specific isoform is regulated by various SFs, leading to tumorigenesis or tumor maintenance [44]. Then, we concentrated on WGCNA to investigate the perturbation of SFs as coordinated networks. Functional enrichment analysis of tumor-associated modules in Fig. 3 revealed that RNA splicing processes like spliceosome, mRNA splicing, and spliceosomal snRNP complex were over-represented in blue, midnightblue, red, and tan modules (Fig. 5c). Co-expressed SFs can be regulated by *MYC* [45]. Interestingly, *MYC*-related biological processes were significantly enriched in most modules (Supplementary Fig. 5d). We found that SFs in tumor-related modules were significantly correlated with *MYC* genes (|r|>0.3, FDR < 0.05) (Supplementary Table S9), of which 85 SFs were hub genes (|GS|>0.5,|MM|>0.5) (Supplementary Fig. 5e). These results show that SFs play important roles in ESCC.

To uncover the responsibilities of dysregulated SFs in regulating AS, we assessed the relationship between the 85 SFs and 3575 DASEs by establishing 303,875 RBP-DASE pairs. First, 49 SFs generally exhibited upregulation with GS and MM > 0 in 2 mRNA and 2 protein cohorts, whereas 36 generally exhibited downregulation with GS and MM < 0. Importantly, we observed that SF expression levels were closely associated with inclusion levels of the corresponding alternative exon. Specifically, both for protein-coding RNA and lncRNAs, most of the DASEs with upregulated PSI showed a negative correlation with downregulated SFs while showing a positive correlation with upregulated SFs in ESCC. Conversely, most DASEs with downregulated PSI showed a negative correlation with upregulated SFs and a positive correlation with downregulated SFs (Fig. 5d). These findings reveal that the 85 hub SFs are crucial to regulating AS in an expression-dependent manner.

To further explore upregulated SFs with clinical relevance, we intersected the genes with prognostic significance (Kaplan-Meier, *p* < 0.05), upregulated expression in the two proteomic cohorts, and upregulated mRNA expression in GSE53625. Finally, we obtained 9 upregulated SFs (Insulin-like Growth Factor 2 mRNA Binding Protein 1 (IGF2BP1), THO Subunit Complex 5 (THOC5), DEK Proto-Oncogene (DEK), RNA Binding Protein With Serine Rich Domain 1 (RNPS1), Non-POU Domain Containing Octamer Binding, (NONO), Heterogeneous Nuclear Ribonucleoprotein A2/B1 (HNRNPA2B1), DExH-Box Helicase 57 (DHX57), Splicing Factor 3B Subunit 2 (SF3B2) and Spliceosome Associated Factor 1 (SART1)) significantly associated with poor prognosis and 4 upregulated SFs (Dead Box Helicase 23 (DDX23), FUS RNA Binding Protein (FUS), Splicing Factor 3B Subunit 4 (SF3B4) and Splicing Factor 3 A Subunit 3 (SF3A3) significantly associated with good prognosis (Fig. 5e). Among the 9 SFs associated with poor prognosis, SF3B2 and DHX57 regulated the most significant number in core and non-core SFs, respectively (Fig. 5f and Supplementary Table S10). SF3B2-regulated DASEs were enriched in the PI3K/AKT/mTOR, Wnt, and ErbB signaling pathways (Fig. 5f and Supplementary Table S11). DHX57-regulated DASEs were enriched in the Hippo, TNF-β, and Wnt signaling pathways (Fig. 5f and Supplementary Table S12). Our results indicate that SFs are crucial in ESCC development by inducing AS.

### SF3B2 promotes ESCC cell proliferation by inducing splicing disruption

**Fig. 6:**
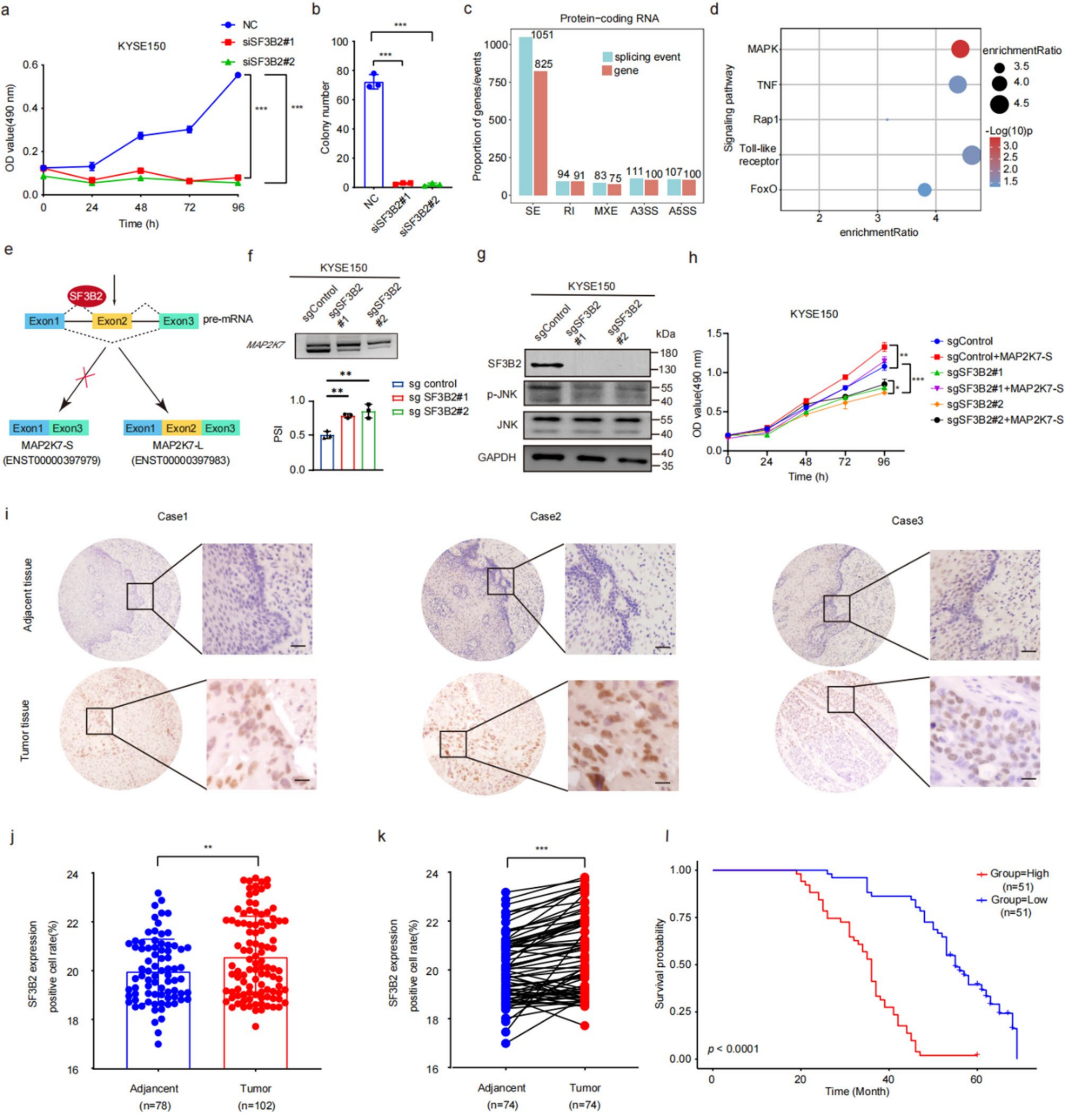
SF3B2 promotes ESCC cell proliferation by inducing splicing disruption. **a** MTT assays conducted in KYSE150 cells with SF3B2 siRNA and a negative control. **b** The clonogenic ability of ESCC cells after siSF3B2 treatment. **c** Bar plot showing the number of the five types of DASEs and affected protein-coding RNA after SF3B2 knockout in the KYSE450 cells. **d** KEGG enrichment analysis of downregulated genes after SF3B2 knockout. **e** Schematic diagram showing that the knockout of SF3B2 leads to increased expression of the long MAP2K7 transcript (MAP2K7-L) and decreased expression of the short MAP2K7 transcript (MAP2K7-S). **f** RT-PCR analysis of MAP2K7 splicing changes in sgSF3B2 KYSE150 cells. **g** The protein expression levels of p-JNK and JNK were measured by Western blot in sgSF2B2 KYSE150 cells. **h** Cell proliferation efficiency was assessed following the recovery of MAP2K7-S isoform into sgSF3B2 KYSE150 cells. **i** Representative IHC images of ESCC tissue array stained with SF3B2 antibody in adjacent and paired ESCC tissues. Scale bar: 50 μm. **j-k** Quantitative analysis of SF3B2 expression in unpaired (**j**) and paired (**k**) ESCC and adjacent tissue according to SF3B2 positive cell rate. **l** Kaplan-Meier survival analysis comparing the prognosis of patients with high and low SF3B2 expression.

Next, we validated SF3B2-related AS events and the function of SF3B2. We initially identified that SF3B2 potentially regulated 490 DASEs, of which SE ranked the priority (Supplementary Fig. 6a). In addition, SF3B2 was positively and negatively correlated with plenty of DASEs in ESCC (Supplementary Fig. 6b). SF3B2 was also significantly upregulated in ESCC cell lines including KYSE150, KYSE180 and KYSE450 by the DepMap CRISPR database (https://depmap.org/*)* [46] (Supplementary Fig. 6c). In order to experimentally verify the function of SF3B2, we evaluated the SF3B2 protein expression in ESCC cells and found that SF3B2 was high particularly in KYSE140, KYSE150, KYSE410, and KYSE450 in comparison to SHEE cells, while KYSE70 exhibited comparatively lower expression (Supplementary Fig. 6d). Subsequently, we knocked down SF3B2 in KYSE150 by siRNA (Supplementary Fig. 6e). Following this, we assessed the dependency of SF3B2 within ESCC and 5 other types of cancer using the genome-wide CRISPR-Cas9 loss-of-function data retrieved from the DepMap CRISPR database. The results revealed that knocking out SF3B2 significantly inhibited cell proliferation in various types of cancer (Supplementary Fig. 6f). As expected, the substantial effect of SF3B2 on the viability of ESCC was validated through MTT assays (Fig. 6a). The plate cloning assay further confirmed a reduction in clonogenicity after siSF3B2 treatment (Fig. 6b and Supplementary Fig. 6g). These findings suggest that SF3B2 maybe play an essential role in the proliferation of ESCC cells by modulating AS.

To further explore the molecular mechanism of SF3B2 promoting the proliferation of ESCC cells by inducing aberrant splicing, we performed knockout of SF3B2 in the ESCC cells and conducted RNA-seq analysis. The results revealed that knockout of SF3B2 led to 1051 aberrant splicing events affecting 825 genes in SE, 94 events affecting 91 genes in RI, 83 events affecting 75 genes in MXE, 111 events affecting 100 genes in A3SS, and 107 events affecting 100 genes in the A5SS type (|∆PSI| >0.1, FDR < 0.05) (Fig. 6c). Morover, KEGG pathway enrichment analysis of downregulated genes (Log_2_FoldChange < −1, FDR < 0.05) indicated significant activation of the MAPK signaling pathway (Fig. 6d). These results suggest that SF3B2 may promote ESCC cell proliferation by activation of the MAPK signaling pathway.

To verify this hypothesis, we focused on mitogen-activated protein kinase kinase 7 (MAP2K7), a key component of the MAPK signaling pathways. Our data showed that SF3B2 knockout facilitates retention of exon 2 in MAP2K7, resulting in increased expression of the long isoform MAP2K7-L and decreased expression of the short isoform MAP2K7-S (Fig. 6e and f). MAP2K7 trigger JNK activity in cells [47]. Moreover, the skipping of exon2 in MAP2K7 is known to form a high-affinity JNK docking site leading to activation of JNK signaling [48]. Our data also demonstrated that SF3B2 knockout led to decreased levels of p-JNK (Fig. 6g). Subsequently, the results revealed that increasing MAP2K7-S expression restored the proliferation ability of sgSF3B2 in a rescue assay (Fig. 6h and Supplementary Fig. 6h). These findings indicate SF3B2 promotes ESCC cell proliferation by inducing the skipping of exon2 in MAP2K7.

To analyze the relationship between SF3B2 expression and clinicopathological features, we performed immunohistochemical staining in the ESCC tissue array. SF3B2 was predominantly localized in the nucleus of ESCC cells (Fig. 6i). SF3B2 was also significantly upregulated in unpaired (Fig. 6j) and paired (Fig. 6k) ESCC tissues compared to adjacent normal tissues. Moreover, high expression of SF3B2 was associated with a worse prognosis, with a median survival time of 36 months in the high-expression group and 55 months in the low-expression group (Fig. 6l; Table 1). After that, we found that SF3B2 was significantly elevated in ESCC and differentially dysregulated among other 8 types of cancer by utilizing samples from the TCGA database (Supplementary Fig. 6i). Taken together, these data indicate that SF3B2 may promote ESCC cell proliferation by regulating the AS.

**Table 1:** The correlation between SF3B2 and clinical characteristics of ESCC patients

Clinicopathological Characteristics		Number (*n* = 102)	Percent (%)	SF3B2		
				High (*n* = 51)	Low (*n* = 51)	
Age	≤ 60	68	66.7	35	33	*p* = 0.674
	> 60	34	33.33	16	18	χ2 = 0.176
Gender	Male	81	79.41	44	37	*p* = 0.087
	Female	21	20.59	7	14	χ2 = 2.938
Pathology grade	I	8	7.84	5	3	*p* = 0.217
	I-II	12	11.76	6	6	
	II	65	63.73	30	35	
	II-III	7	6.86	2	5	
	III	10	9.80	8	2	χ2 = 5.770
T classification	Tis	2	1.96	0	2	*p* = 0.178
	T1-T2	37	36.27	16	21	
	T3-T4	63	61.76	35	28	χ2 = 3.453
lymph node	N0	78	76.47	36	42	*p* = 0.161
	N1	24	23.53	15	9	χ2 = 1.962
Follow-uptime, months		102		36	55	*p* < 0.0001 HR (95% CI)) = 4.352 [2.657, 7.129]

TNM staging of cancer including Tumor (T), Node (N), and Metastasis (M), as defined by the American Joint Committee on Cancer (AJCC) 8th edition

### The drugs associated with splicing are effective in ESCC

**Fig. 7:**
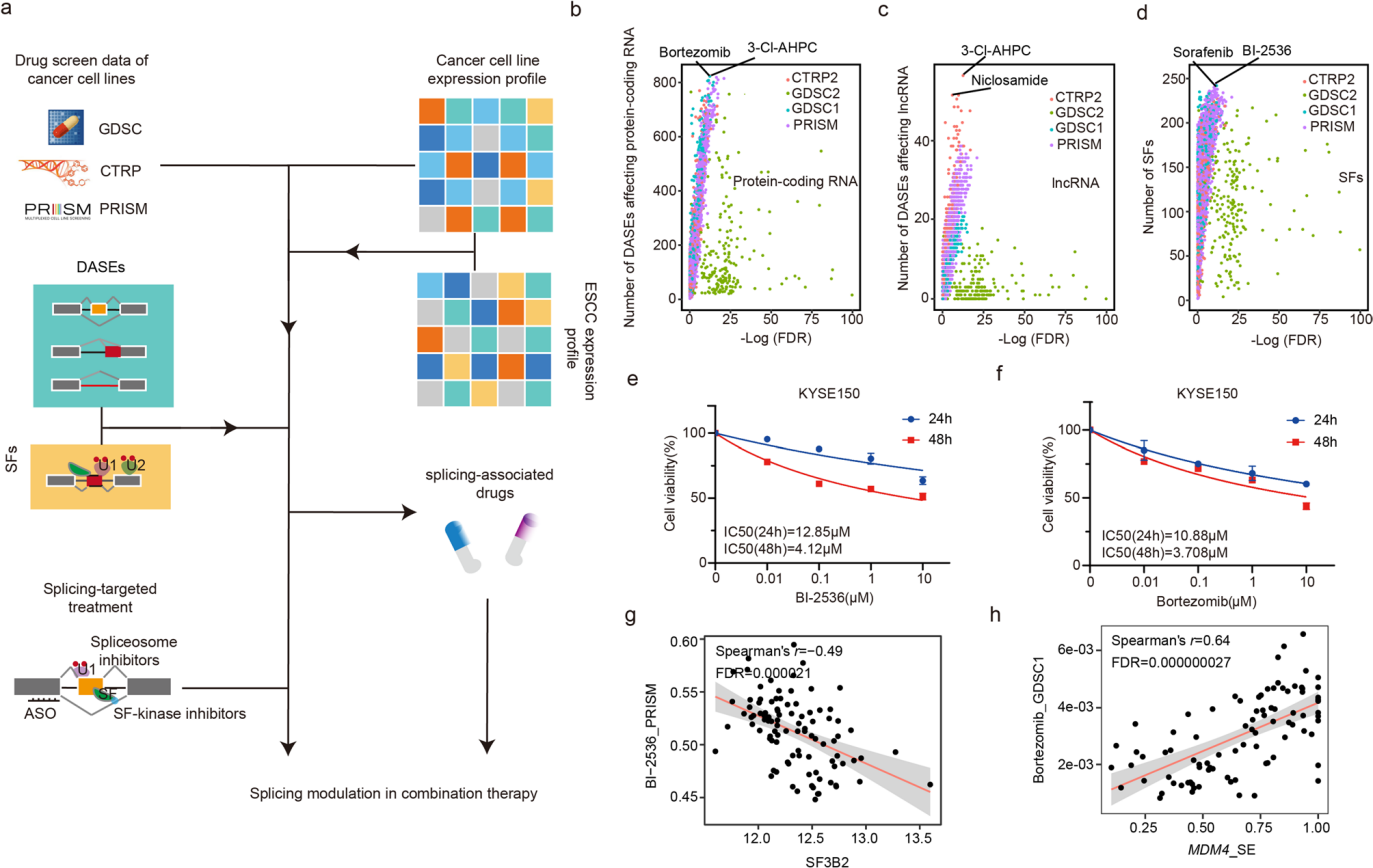
The drugs associated with splicing are effective in ESCC. **a** Flowchart illustrating the pipeline for identifying splicing-related drugs with sensitivity for ESCC. **b-d** Scatter plots showing the number of splicing events affecting protein-coding genes (**b**), lncRNAs (**c**), and SFs (**d**) associated with drugs from four databases. The differences in sensitivity of these drugs between ESCC and adjacent samples were compared. The screening criteria are as follows: FDR < 0.05 and log_2_(FoldChange) < −0.1. X-axis: -Log_10_ (FDR) of drug sensitivity between ESCC and adjacent tissues. Y-axis: Number of DASEs and SFs associated with drugs. **e**,** f** Cytotoxicity assay of KYSE150 cells treated with BI-2536 (**e**) and Bortezomib (**f**) at range of concentrations for 24 and 48 h. **g** Scatter plot showing the correlation between SF3B2 expression levels and BI-2536 sensitivity response level. **h** Scatter plot depicting the correlation between inclusion levels of *MDM4* and response levels to Bortezomib.

Dysregulation of AS can hinder chemotherapy efficacy [49]. To characterized clinical relevance of the spicing, the splicing-associated drugs were screened using the pipeline depicted in Fig. 7a. Among the 525 screened drugs, 3-Cl-AHPC and Bortezomib were found to be significantly associated with the most of protein-coding RNA events with 825 and 823, respectively (Fig. 7b), while 3-Cl-AHPC and Niclosamide were significantly associated with the majority of lncRNA events with 57 and 52, respectively (Fig. 7c). Drugs BI-2536 and Sorafenib were significantly correlated with 243 and 242 SFs, respectively (Fig. 7d and Supplementary Table S13). To experimentally validate the effect of the screened drug, we performed cytotoxicity assay. BI-2536 showed low IC50 at 24 h with 12.85 µM and 48 h with 4.12 µM (Fig. 7e), while Bortezomib showed 10.88 µM at 24 h and 3.708 µM at 48 h (Fig. 7f). Moreover, we found that high sensitivity to BI-2536 was associated with high expression of SF3B2 (Fig. 7g), and high sensitivity to Bortezomib was found to be correlated with the skipping of exon 6 in *MDM4* (Fig. 7h). This data indicated that targeting protein-coding RNA events or SFs were effective. Together, we identify a range of drugs sensitive to ESCC, which exhibit correlations with DASEs and SFs in ESCC.

## Discussion

**Fig. 8:**
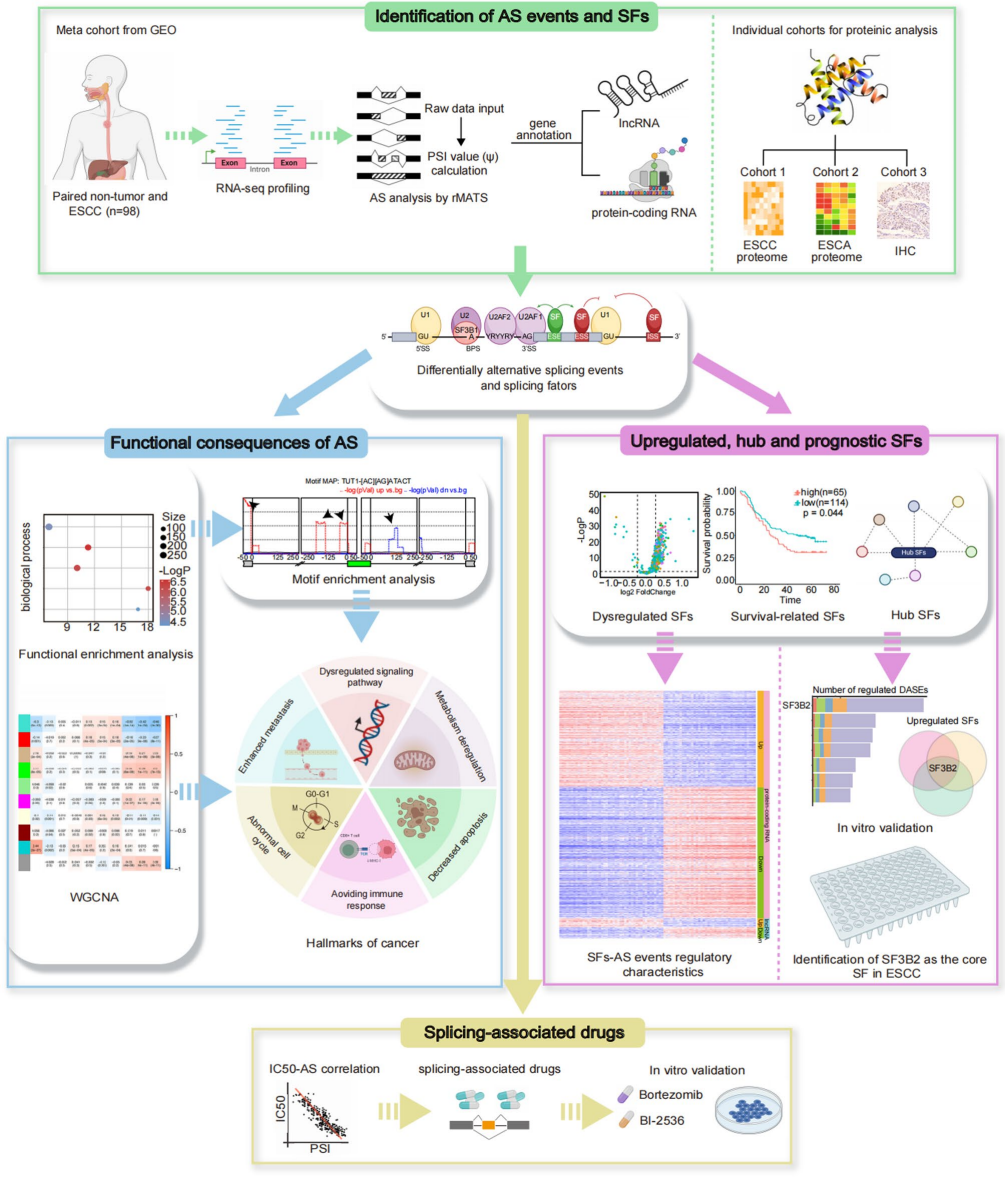
Flow chart indicating the study design. ESCC data obtained from GEO database was used to perform rMATS analysis. Two proteomic cohorts were used to analyze expression of SFs. WGCNA and motif enrichment analyses reveal the biological roles of DASEs and SFs. Spearman correlation analyses with DASEs were implemented for drug discovery.

The dysregulation of AS plays a vital role in regulating the occurrence and development of various cancers [11–14]. A clear insight into AS in cancer may promote our understanding of tumor pathogenesis and nominate several novel therapeutic strategies. Here, we profiled a comprehensive repertoire of AS in ESCC and deeply explored the regulatory characteristics of SFs, downstream biological processes, and splicing-associated drugs (Fig. 8). Our study first investigated the landscape of AS and SFs from multiple perspectives by transcriptomic and proteomic datasets, which facilitates the understandings of molecular characteristics and promotes precision medicine in ESCC.

LncRNAs are more abundant than protein-coding RNAs in the genome and regulate gene expression modulating signaling pathways [50]. Emerging evidence has shown that lncRNAs can act as products of AS, playing a role in promoting or suppressing cancer [51]. The retention of the intron 3 of *PXN-AS1* promotes hepatocellular carcinoma metastasis by inducing *MYC* transcription activation [42]. LncRNAs may be used as prognostic and early diagnostic biomarkers for ESCC [52], and detected ample abnormal AS events affecting lncRNAs in ESCC [15, 53]. In our study, we mapped lncRNA splicing landscape and determined their biological effects by analyzing the functional correlation with protein-coding RNAs, as well as probing their upstream SFs, providing further understanding of AS in ESCC.

The absence of high-throughput methods to examine the function of spliced isoforms hinders the further exploration of biological roles for AS [9]. In this study, we utilized WGCNA to elucidate the global functional consequences of AS in ESCC, rather than focusing on a single tumor suppressor or oncogene. WGCNA discovers potential functions of genes without functional annotation information and identifies modules with specific biological processes or cell-type-specific effects [54–56]. Unlike traditional correlation analyses that evaluate the AS functions through pathway activity, such as pathway enrichment-guided activity study of alternative splicing (PEGASAS) [11], single sample gene set enrichment analysis (ssGSEA) [13], and gene set variation analysis (GSVA) [57], our approach focused on AS-module analysis. This framework enabled us to view functional genes associated with AS as interconnected units, facilitating the exploration of AS in relation to various traits or phenotypes. Our findings indicated that AS in ESCC impacts abnormal signaling pathways, the cell cycle, immune response, metastasis, apoptosis, and metabolism. Each ME was significantly associated with 545 to 958 AS events, suggesting a potential relationship between AS events and hallmarks of cancer. This approach enhances our understanding of the biological implications of AS events.

AS is modulated by sequence-specific RBPs that function in a cell-, tissue-, and subtype-specific fashion as both oncoproteins and tumor suppressors [12, 58–60]. By inducing splicing alterations affecting cancer-related hallmarks, SFs can potentially disrupt a network of gene products and cancer pathways [9]. Our data revealed that PABPC5 and RBM6 were involved in metastasis, RBM42 was involved in metabolism, CNOT4 was involved in the cell cycle, TUT1 was involved in immune response, and SNRNP70 was involved in apoptosis. Dissection based on the relationship between splicing factors and downstream genes and AS events featured with functional implications also showed significant specificity. Several of the SFs identified herein have previously been reported in previous research. Mutation of PABPC5 was identified to be correlated with NSCLC metastasis [61]. CNOT4 promotes NSCLC proliferation by facilitating the ubiquitination of PAF1 [62]. TUT1 promotes Talaromyces marneffei to escape macrophage killing via mediating alternative splicing of NCOR2/SMRT [63]. SF3B2-mediated RNA splicing derives the prostate cancer progression [64, 65]. In this study, we found that SF3B2 promotes ESCC proliferation by disturbing AS profiles. These indicate that SF3B2 may be a promising therapeutic target in ESCC. Moreover, the biological roles will be explored in the further experiments, providing valuable insights into the functional associations between SFs and cancer-related phenotypes.

Screening for drugs that inhibit tumor progression helps combat cancer and address drug resistance [66, 67]. AS is proven to perturb the expression of a wide range of relevant genes participating in drug metabolism and activating signaling pathways [49]. Conceivably, tumor exploits the abnormal AS to evade the efficacy of cytotoxic drug treatment and contribute to drug resistance. Thus, discovering novel splicing-associated drugs is crucial for improving treatment outcomes. Here, Bortezomib and BI-2536 effectively inhibited ESCC cell proliferation. The sensitivity of Bortezomib was positively correlated with the inclusion of exon 6 in *MDM4.* Interestingly, ASO-mediated *MDM4* exon 6 skipping impedes tumor growth [22], indicating Bortezomib may inhibit the proliferation of ESCC by targeting AS. Therefore, our research provides the potential theory that targeting splicing modulators may expand the therapeutic strategies for ESCC. It also highlights the promising prospects for the combination with splicing-targeted therapy.

## Conclusion

In summary, our research mapped the landscape of AS and SF dysregulation in ESCC, explored its related biological implications, and proposed combined drug treatment strategies. This study deepens our understanding of the molecular characteristics of ESCC, contributes to the development of innovative treatments, and may promote basic research on precision medicine for ESCC.

## Supplementary Information

Supplementary material 1

Supplementary material 2

Supplementary material 3

Supplementary material 4

Supplementary material 5

Supplementary material 6

Supplementary material 7

Supplementary material 8

Supplementary material 9

Supplementary material 10

Supplementary material 11

Supplementary material 12

Supplementary material 13

Supplementary material 14

Supplementary material 15

Supplementary material 16

Supplementary material 17

Supplementary material 18

Supplementary material 19

## Data Availability

Raw RNA-seq data can be obtained from Gene Expression Omnibus (GEO) database (PRJNA533799, PRJNA435587, PRJNA704753, PRJNA489250, PRJNA298963) for AS analysis. The microarray dataset can be collected from GSE53625. The raw proteomics data can be obtained in the iProX database (IPX0002466000) and PRIDE database (PXD021701). The Cancer Genome Atlas (TCGA) data was downloaded from UCSC Xena (https://xena.ucsc.edu/welcome-to-ucsc-xena/).

## Abbreviations

ESCC: Esophageal squamous cell carcinoma
AS: Alternative splicing
SF: Splicing factor
DASE: Differential alternative splicing event
lncRNA: Long non-coding RNA
RBP: RNA-binding protein
TCGA: The cancer genome atlas
GEO: Gene expression omnibus
GO: Gene-ontology
KEGG: Kyoto encyclopedia of genes and genomes
GSEA: Gene set enrichment analysis
WGCNA: Weighted correlation network analysis
IC50: Half-maximal inhibitory concentration
SF3B2: Splicing factor 3B subunit 2
MAP2K7: Mitogen-activated protein kinase kinase 7
