# Supplementary figures and images for: Global landscape of protein-coding and long non-coding RNA alternative splicing and regulation in the human esophageal squamous cell carcinoma
Source: Cancer Cell Int. 2026 Jun 22;26:236. doi: 10.1186/s12935-025-03921-9 (PMC13285186; doi:10.1186/s12935-025-03921-9)

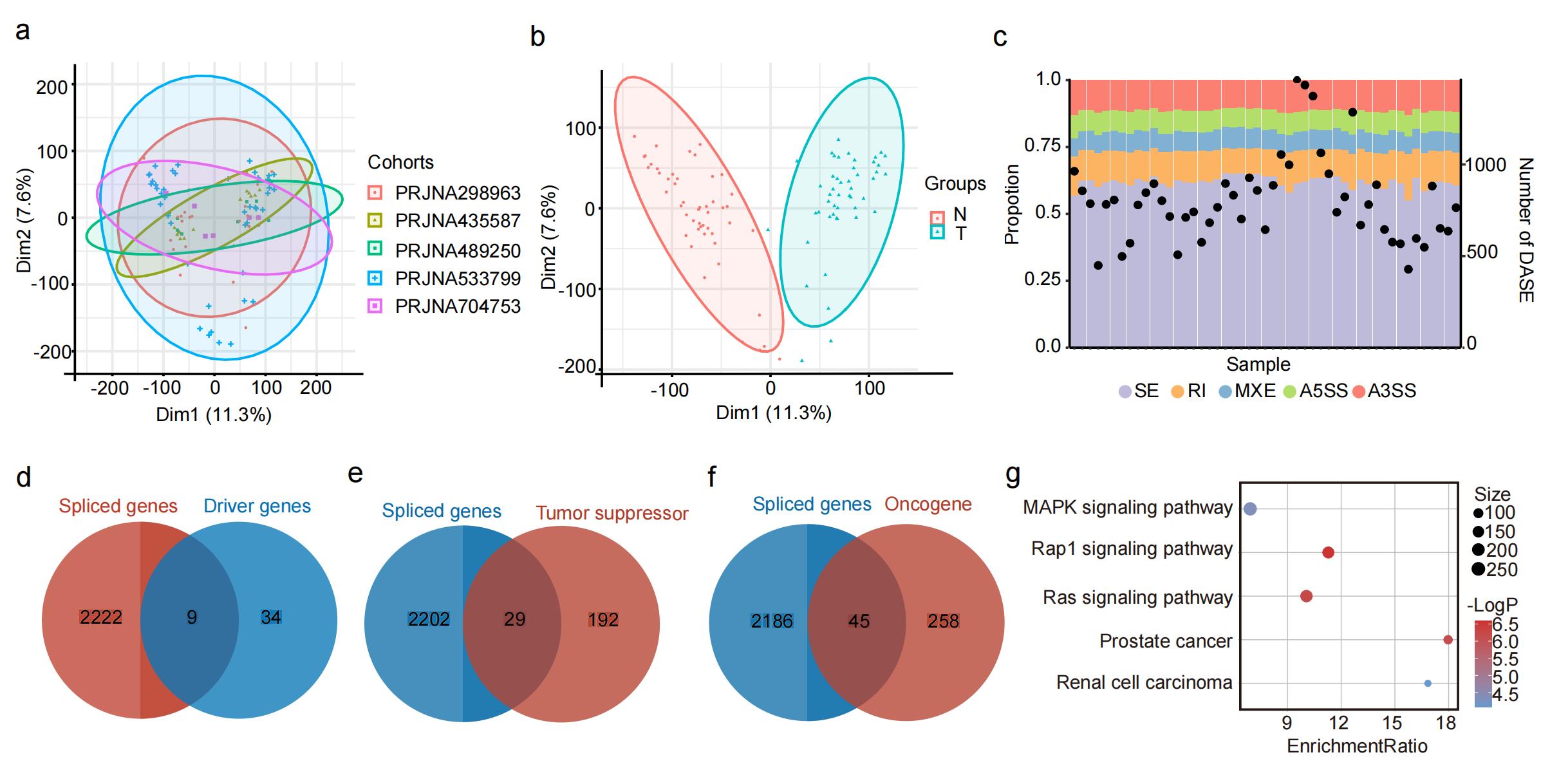

Supplement: Supplementary file 1 — Supplementary material 1 [file 12935_2025_3921_MOESM1_ESM.jpg]

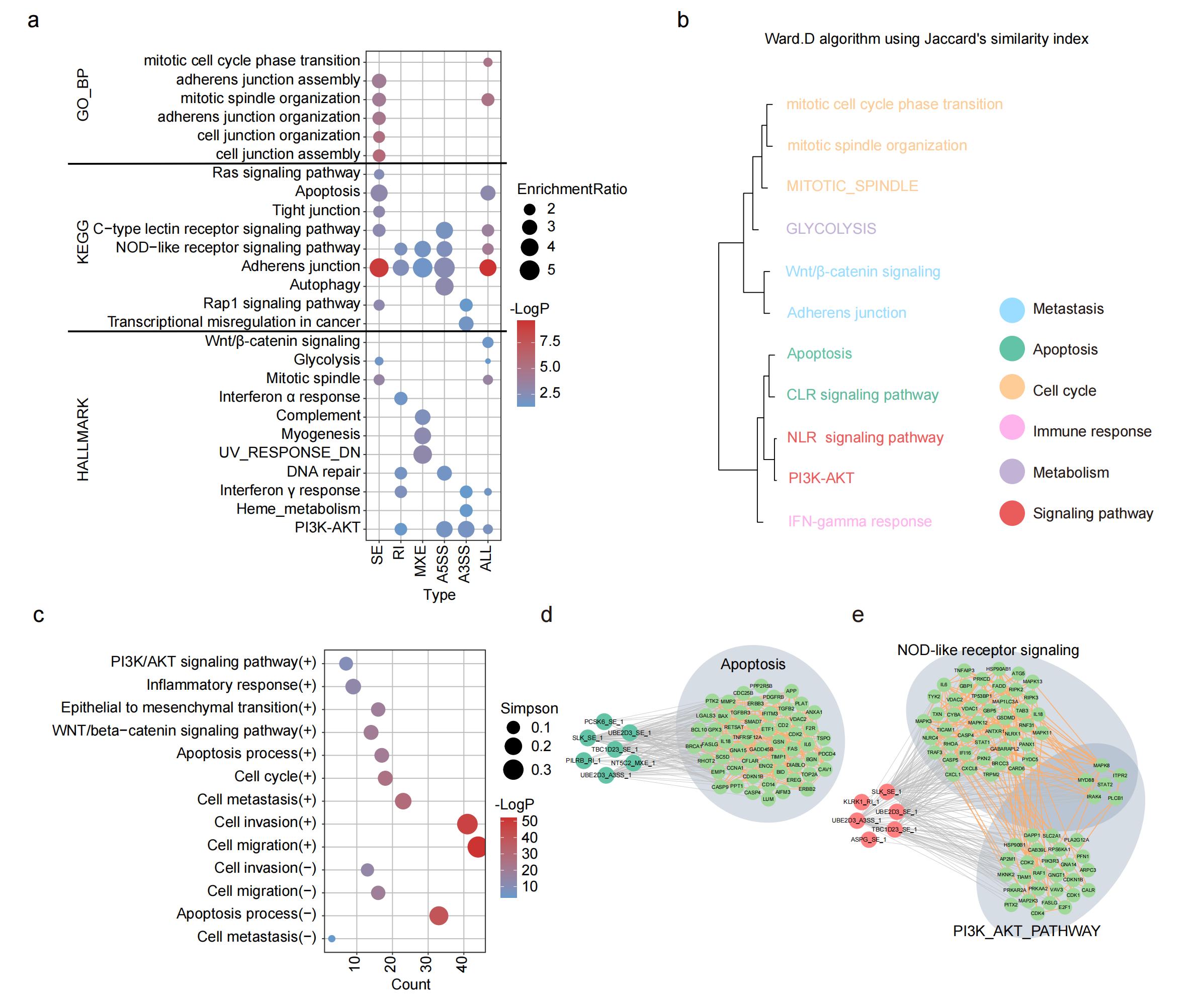

Supplement: Supplementary file 2 — Supplementary material 2 [file 12935_2025_3921_MOESM2_ESM.jpg]

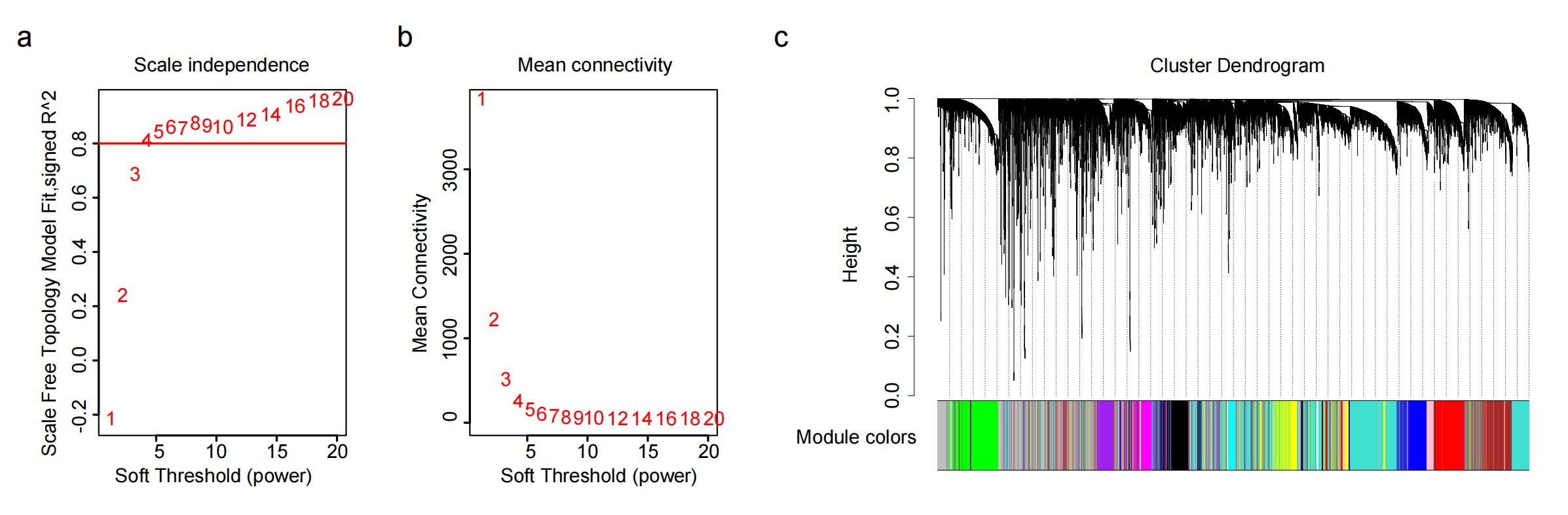

Supplement: Supplementary file 3 — Supplementary material 3 [file 12935_2025_3921_MOESM3_ESM.jpg]

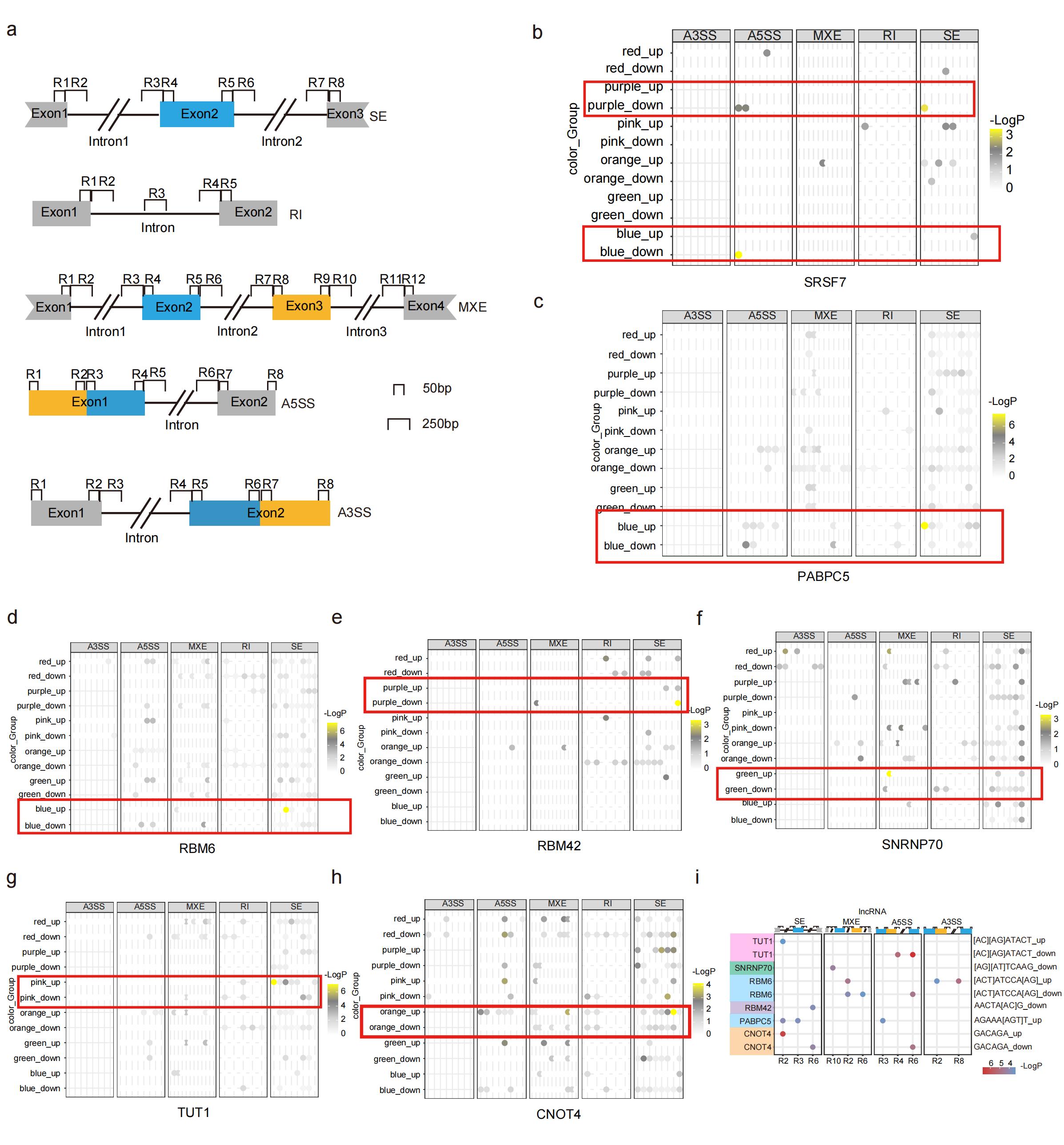

Supplement: Supplementary file 4 — Supplementary material 4 [file 12935_2025_3921_MOESM4_ESM.jpg]

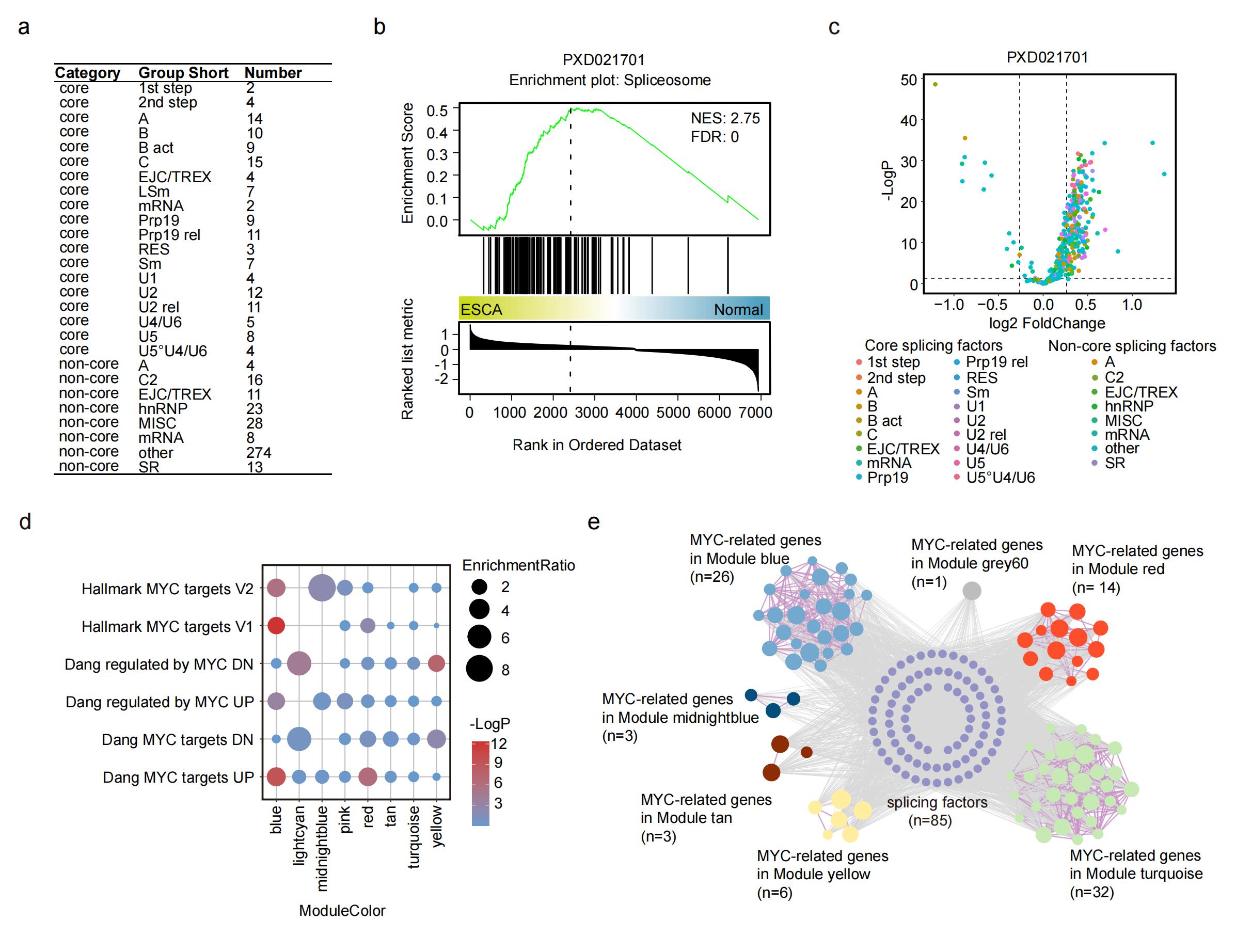

Supplement: Supplementary file 5 — Supplementary material 5 [file 12935_2025_3921_MOESM5_ESM.jpg]

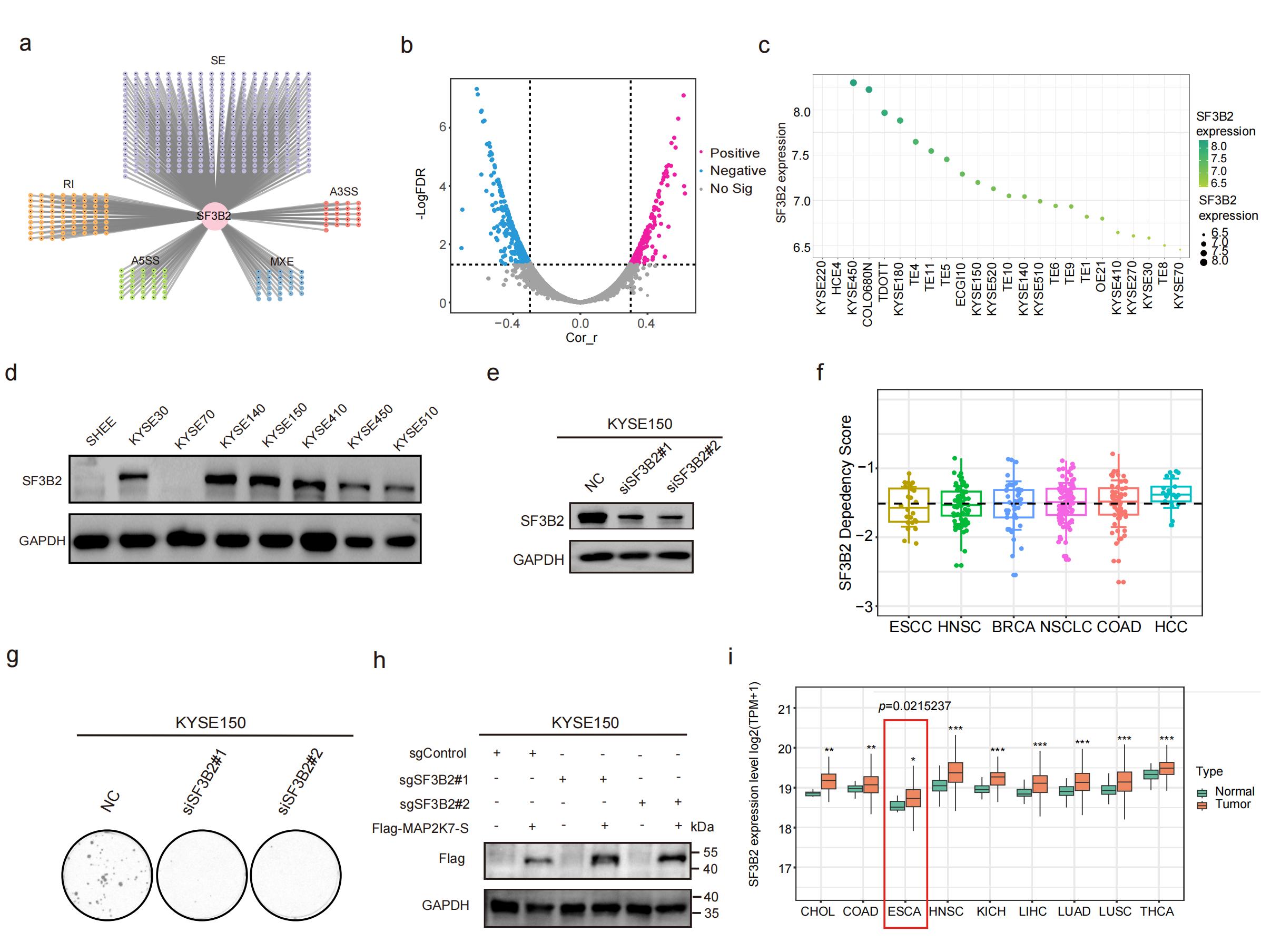

Supplement: Supplementary file 6 — Supplementary material 6 [file 12935_2025_3921_MOESM6_ESM.jpg]
